# A hardware Markov chain algorithm realized in a single device for machine learning

**DOI:** 10.1038/s41467-018-06644-w

**Published:** 2018-10-17

**Authors:** He Tian, Xue-Feng Wang, Mohammad Ali Mohammad, Guang-Yang Gou, Fan Wu, Yi Yang, Tian-Ling Ren

**Affiliations:** 1 Institute of Microelectronics, Tsinghua University, Beijing, 100084 China; 2 Beijing National Research Center for Information Science and Technology (BNRist), Tsinghua University, Beijing, 100084 China; 3 School of Chemical and Materials Engineering (SCME), National University of Sciences and Technology (NUST), Sector H-12, Islamabad, 44000 Pakistan

## Abstract

There is a growing need for developing machine learning applications. However, implementation of the machine learning algorithm consumes a huge number of transistors or memory devices on-chip. Developing a machine learning capability in a single device has so far remained elusive. Here, we build a Markov chain algorithm in a single device based on the native oxide of two dimensional multilayer tin selenide. After probing the electrical transport in vertical tin oxide/tin selenide/tin oxide heterostructures, two sudden current jumps are observed during the set and reset processes. Furthermore, five filament states are observed. After classifying five filament states into three states of the Markov chain, the probabilities between each states show convergence values after multiple testing cycles. Based on this device, we demo a fixed-probability random number generator within 5% error rate. This work sheds light on a single device as one hardware core with Markov chain algorithm.

## Abstract (web summary)

Despite the need to develop resistive random access memory (RRAM) devices for machine learning, RRAM array-based hardware methods for algorithm require external electronics. Here, the authors realize a Markov chain algorithm in a single 2D multilayer SnSe device without external electronics.

## Introduction

In the past decade, we have witnessed a fast growing trend in developing two-dimensional (2D) material-based devices using graphene^1^, MoS_2_^2^, etc. Although the 2D material family is very large; however, limited options^3^ (such as graphene, hBN, MoS_2_, and WSe_2_) exist with moderate stability in ambient conditions. The remaining 2D materials (silicene^4^, metallic dichalcogenides^3^, etc.) show rapid degradation in ambient conditions, which is a major obstacle for practical applications. A critical question is how to develop novel devices based on other 2D materials in ambient conditions. The oxidation of 2D materials is regarded as a drawback, which can introduce scattering sources and trapping centers in the forbidden band resulting in hysteresis^5^, fast degradation^6^, or even decomposition leading to loss of film altogether^3,7^. Another group of 2D crystals are numerous oxides which cover a rich variety of materials such as micas, layered Cu oxides, TiO_2_, layered perovskites, etc.^8–10^. As 2D oxides, these crystals are less sensitive to air but they tend to lose oxygen and may react with water^3^. In order to obtain robust 2D oxides, direct oxidation of 2D materials for fabricating resistive memories have also been used^11,12^. But the electrical behaviors of these materials are similar to that of traditional resistive memories. The native oxide of 2D materials may be potentially useful as a resistive switching layer with different behaviors to traditional resistive memories—an application area which has rarely been investigated.

Resistive random access memory (RRAM) shows great potential for machine learning applications due to its working mechanism involving ionic transport and hence good analogy to bio synapses^13,14^. However, hardware approaches in machine learning applications are mainly based on an array of RRAM devices with external electronics^15,16^, which require software or hardware assistance. At present, using a RRAM array^15,17^ can only implement or built partial algorithms (matrix multiplication and weight update). Besides, although different kinds of machine learning algorithms can be realized by software approaches, it consumes even more transistors on-chip^18^. The machine learning algorithm realized at a single device level remains elusive. If the learning algorithm can be realized in a single device without external electronics, the machine learning system can be extremely compact and efficient. Markov chain is one of the most important and fundamental algorithms in the field of machine learning. This algorithm has wide applications for modeling queueing systems, the internet, remanufacturing systems, inventory systems, DNA sequences, genetic networks, and many other practical systems^19^. For example, in DNA sequences, Markov chain is used to predict the existence of DNA bases (A, C, T, G) at certain sites^20^.

Here, we propose a Markov machine learning algorithm in a single device based on native tin oxide (SnO_*x*_) of multilayer tin selenide (SnSe). The SnO_*x*_/SnSe/SnO_*x*_ stack is a self-formed heterostructure by creating native oxide on both sides of the SnSe at room temperature (300 K), in air (21% oxygen) for 24 h. The thickness of native oxide SnO_*x*_ via transmission electron microscope (TEM) for both thin (~40 nm) and thick SnSe (~200 nm) are around 10 nm, which is a further indication of the self-limited mechanism of native oxide in SnSe. The native oxide SnO_x_ thickness is self-limited, which can guarantee repeatable resistance states despite different total SnSe thickness. Anomalous double set and reset processes are observed during unipolar switching. The theoretical model is also established and double set and reset phenomenon can be well understood, which can be connected to Markov chain algorithm. We also demo a single SnO_*x*_/SnSe/SnO_*x*_ device as one core of Markov chain and the probabilities between each states show convergence values after multiple testing cycles.

## Results

### Characterization of SnSe-RRAM

**Fig. 1:**
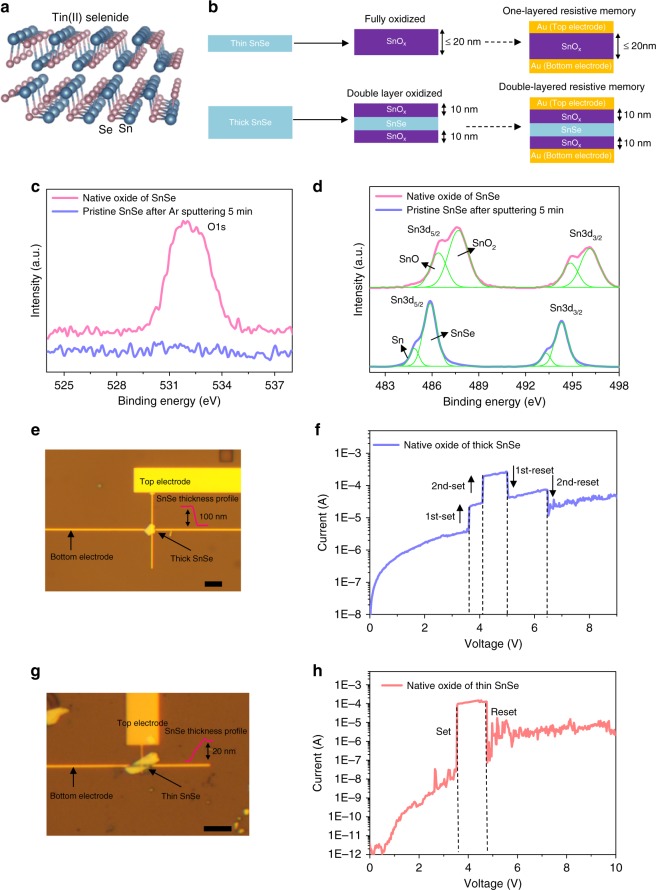
Resistive memory based on native oxide of SnSe. **a** Basic orthorhombic crystal structure of SnSe. **b** The schematic of SnSe oxidation process for two different thickness results in SnO_*x*_ resistive memory and SnO_x_/SnSe/SnO_*x*_ resistive memory. The X-ray photoelectron spectroscopy (XPS) results of native oxide of SnSe and pristine SnSe for (**c**) O and (**d**) Sn peaks. The native oxide of SnSe is the mixture of SnO_2_ and SnO. **e** The optical image and atomic force microscope (AFM) thickness profile for a 100 nm SnO_*x*_/SnSe/SnO_x_ device. The scale bar is 5 μm. **f** Typical switching behavior of SnO_*x*_/SnSe/SnO_*x*_ resistive memory with distinct two-step set and reset processes. **g** The optical image and AFM thickness profile for a 20 nm SnO_*x*_ device. The scale bar is 5 μm. **h** Typical switching behavior of SnO_*x*_ based resistive memory with regular one set and reset processes

Recently, black phosphorus with orthorhombic crystal structure (Cmca space group) attracted a lot of attention due to its high mobility, anisotropy, large dispersion of bandgap by tuning layer numbers, etc.^21^. Meanwhile, there is another orthorhombic crystal SnSe at room temperature in Pnma space group. Its bulk form shows high-ZT factor^22,23^, which makes it a suitable candidate for being used in a wide variety of thermo-electrical devices. However, the 2D form of the multilayer SnSe is relatively unexplored. Here, we investigate SnSe thin films by mechanical exfoliation in ambient conditions. Figure 1a shows the orthorhombic crystal structure of SnSe. Similar to black phosphorus, SnSe has strong anisotropy due to the effective mass difference in armchair and zigzag directions. The in-plane Sn–Se covalent bonds exhibit puckered structure and the SnSe layers are bonded by weak van der Waals forces. As a result, SnSe flakes can be obtained by mechanical exfoliation. Pristine SnSe flake observed by TEM shows an interlayer distance of 1.325 nm and the diffraction image indicates a high-quality crystal structure (see Supplementary Fig. 1). The oxidized SnSe is also observed by the TEM cross-section with sandwich structure of native oxide (10 nm)/SnSe/native oxide (10 nm) (see Supplementary Fig. 2). As shown in Fig. 1b, two types of resistive memory devices are proposed based on the thickness of SnSe. For thin SnSe films (≤20 nm), due to the complete SnSe oxidation in air, a conventional metal–oxide–metal device can be obtained. While for thick SnSe films, only top and bottom surfaces are oxidized and thus we can create a novel metal-oxide-SnSe-oxide–metal structure (Fig. 1b). This novel structure may have anomalous resistive behaviors and is worth exploring. The binding energy of native oxide and SnSe were analyzed by X-ray photoelectron spectroscopy (XPS). The large-area exfoliated SnSe flakes exposed in air (24 h) shows a very strong O1s peak, while the pristine SnSe sample after Ar sputtering (5 min) shows almost no O1s peak (Fig. 1c). This indicates that the native oxide is only present at the surface. Moreover, the SnSe with native oxide on the surface exhibits a noticeable double peak in Sn3d_5/2_ and Sn3d_3/2_, which corresponds to the mixture of SnO_2_ and SnO (Fig. 1d). The intrinsic SnSe after Ar sputtering shows a main peak of SnSe with a small reduction in the peak height of Sn. In this way, we can conclude that the native oxide is SnO_*x*_ (*x* = 1 or 2).

Since the thicker SnSe can form native oxide on both sides of SnSe, the SnO_*x*_/SnSe/SnO_*x*_ heterostructure can be used as a resistive memory. A cross-bar (500 nm × 500 nm) based on bottom and top Au electrodes sandwich the SnO_*x*_/SnSe/SnO_*x*_ heterostructure. This minimizes the effective device area and improves the device uniformity (see Methods for detail fabrication process). A typical 100 nm SnSe sample is shown in Fig. 1e. The initial SnSe is quite resistive (~100 MΩ) due to 10 nm oxidation on both sides. After the forming at 6.7 V (see Supplementary Fig. 3) the SnO_*x*_/SnSe/SnO_*x*_ heterostructures become conductive (~100 kΩ). Meanwhile, the intrinsic SnSe with 40 nm thickness shows a resistance of ~500 Ω in the vertical direction (Supplementary Fig. 4). A typical switching behavior for SnO_*x*_/SnSe/SnO_*x*_ heterostructures is shown in Fig. 1f. There are obvious two-step upward jumps in current at 3.6 V (first-set) and 4.1 V (second-set) and two-step downward jumps at 5.0 V (first-reset) and 6.5 V (second-reset). The reproducibility has been demonstrated among more than 20 samples with the yield around 80%. Such behavior is quite different from conventional resistive memory with single set and reset process. Also our device behavior is different from complimentary resistive switches (CRS)^24^, which contains two antiserial RRAM devices with bipolar switching. In our device, two in-serial resistive memory devices are formed with unique two-step set and two-step reset. Since the multi-layer SnSe material is in the middle, our device tends to operate with unipolar switching. This unique behavior (two-step set and two-step reset) cannot be realized by CRS due to the antiserial connection and bipolar switching of that device. Another fully oxidized SnSe sample with ~20 nm thickness is also fabricated (Fig. 1g). It shows a regular single set and reset at 3.5 and 4.8 V (Fig. 1h), respectively. The set and reset voltage are quite close to the value of first-set and first-reset in Fig. 1e. This indicates that the upper and lower oxide layer in the SnO_*x*_/SnSe/SnO_*x*_ heterostructure sets and resets separately due to the SnSe in the middle. This is in contrast to the fully oxidized SnO_x_, which can form a filament as a result of only one set step as no blocking layer (of SnSe) is in the middle. The reverse voltage sweeps immediately after the forward sweeps can induce the unwanted set and influence the cycle to cycle reproducibility (Supplementary Fig. 5). In order to keep stable cycling performance, only single sweep from 0 to 10 V was applied to the devices.

### Double set and reset behaviors of SnO_*x*_/SnSe/SnO_*x*_ heterostructure

**Fig. 2:**
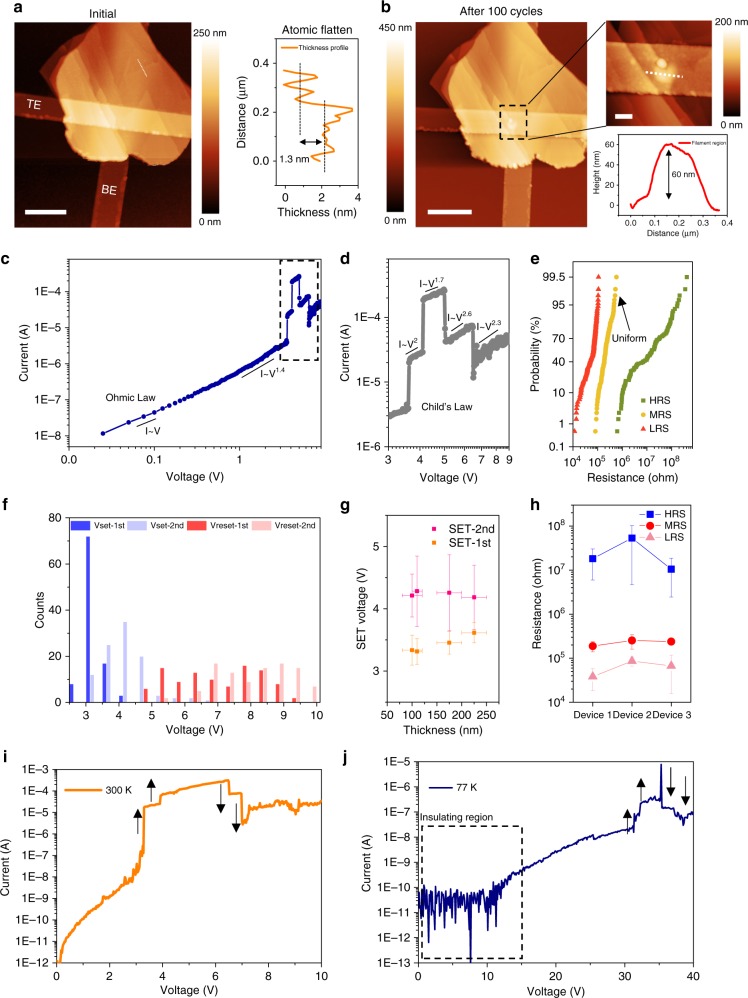
Resistive switching behavior based on SnSe RRAM. **a** AFM results showing the surface of SnO_*x*_/SnSe/SnO_*x*_ with atomic flatness. Both top electrode (TE) and bottom electrode (BE) are shown. The scale bar is 1 μm. **b** AFM results showing generation of bubbles in filament region after 100 cycles operation. The scale bar is 1 μm. The inset shows the zoom-in image of AFM and the related thickness profile. The scale bar of inset AFM image is 200 nm. **c** The resistive switching behavior plotted in log-scale showing that the space-charge-limited mechanism dominates the electrical transport. The voltage region is fit with Ohmic law. **d** The zoom-in image of the dashed line in Fig. 3c showing the Child’s law relation at high voltage region. **e** The HRS, MRS, and LRS distribution. **f** The first, second set, and reset voltage distribution. **g** The first, second set voltage vs. SnSe total thickness. The error bars in *y*-direction are calculated based on the set voltage in 100 cycles for each device. The error bars in *x*-direction are calculated based on the thickness variations from AFM results for each SnSe flake. **h** The HRS, MRS and LRS in three devices with ~100 nm thickness. The error bars for HRS, MRS, and LRS are calculated from 100 cycles for each device. The two-step set/reset switching behavior of SnO_*x*_/SnSe/SnO_*x*_ resistive memory under (**i**) 300 K and (**j**) 77 K, respectively

The surface of SnO_*x*_/SnSe/SnO_*x*_ heterostructures is investigated by an atomic force microscope (AFM). The sample surface still shows atomic steps with roughness less than 1.3 nm (Fig. 2a). Such a phenomenon is drastically different from conventional native oxide (such as AlO_*x*_) which exhibits an obvious surface roughness. The surface of oxidized SnSe is quite flat, which is suitable for resistive memory with improved uniformity. After 100 switching cycles, an obvious bubble is observed (Fig. 2b) with 60 nm height in the center, which could be explained by the oxygen accumulation in the filament region near the top electrode. Such behavior indicates that the filament type could be related to the oxygen vacancies due to the loss of oxygen ions^25^.

The SnO_*x*_ can be regarded as an insulator where the injected electrons can easily exceed the equilibrium concentration and fill the trap states in the forbidden band. As a result, the conduction mechanism of SnO_*x*_/SnSe/SnO_*x*_ heterostructure can be explained by space-charge-limited current (SCLC)^26^, which describes the conduction in the insulator with traps. The SCLC is related to the trap-filled process, which will promote the growth of current and result in steeper curve under larger bias. Based on the SCLC theory, linear dependence with voltage at low voltage (0–1 V), as shown in Fig. 2c, is related to the Ohmic law region: *I* ∝ *V*. When the voltage is in the range of 3.6–4.2 V, current follows a squared dependence with voltage *I* ∝ *V*^2^, which is related to Child’s law region. In this region, the free electrons exceed the equilibrium concentration in the SnO_*x*_ and fill the trapped states, resulting in a large contribution to the current. When the voltage continues increasing past 4.2 V, the high-electrical field described by the Frenkel effect can lower the trap depth inside the SnO_*x*_, and make the electrons jump out to be conducted easier, which can contribute to increasing the index factor larger than two (Fig. 2d). The other conduction mechanisms can be excluded, e.g., Poole–Frenkel (PF) emission requires a linear relationship ${\mathrm{In}}\left( {{\mathrm{I/V}}} \right) \propto \sqrt V$, and Schottky emission requires another type of linear relation ${\mathrm{In}}\left( {\mathrm{I}} \right) \propto \sqrt V$. The PF or Schottky emission is related to the electrode/oxide interface limited mechanism, while the SCLC indicates that the SnO_*x*_/SnSe/SnO_*x*_ heterostructure dominates the conduction instead of the electrode/oxide interface. Three more curves have been fitted (Supplementary Fig. 6). All of the results show similar trend of Ohmic law at low voltage bias and Child’s law at high-voltage bias, which belong to the SCLC theory. The initial resistance under low voltage is in the high-resistance state (HRS). After the first-set, the device is in the middle-resistance state (MRS). After the second-set, the device is in the low-resistance state (LRS). The HRS, MRS, and LRS distribution are shown with good uniformity (Fig. 2e). Especially, the MRS shows quite stable resistance level around 200 kΩ. This proves that the MRS is a physically stable state in the SnO_*x*_/SnSe/SnO_*x*_ heterostructure without controlling the compliance current. The distribution of first- and second-set and reset voltage are shown in Fig. 2f. The first- and second-set are always centered at 3.0 V and 4.2 V, respectively with Gaussian distribution. The resistive switching performance is also verified with different SnSe total thickness ranging from 75 to 250 nm. Different thickness devices show similar first- and second-set voltage (Fig. 2g) at 3.3–3.6 V and 4.2–4.3 V, respectively. This indicates that the oxide layer on both sides of the SnSe is dominated by the electrical conduction and the native oxide thickness is self-limited with similar thickness. Comparing to the set voltage vs. thickness in Fig. 2g, reset voltage vs. thickness (Supplementary Fig. 7) has larger voltage distributions. This is due to the dynamic and competing effects between filament growth and filament rupture during the reset process^27^. Not only is the set voltage similar, the HRS, MRS and LRS are also uniform among three devices with ~100 nm thickness (Fig. 2h). Especially, the MRS state always shows resistance around 200 kΩ among different devices.

In order to understand the anomalous switching with double set and reset behaviors, the SnO_*x*_/SnSe/SnO_*x*_ heterostructure is switched under 300 and 77 K. Under 300 K, it shows similar double-jump behavior (Fig. 2i). However, after the temperature cools down to 77 K (Fig. 2j), the SnO_*x*_/SnSe/SnO_*x*_ heterostructure shows an insulating region when the voltage is swept from 0 to 15 V. The first set occurs at a relatively large voltage of 31.3 V and second set at 32.2 V, followed by first-reset at 35.4 V and second-reset at 37.2 V. Both set and reset voltage increase obviously when compared to set/reset voltages at 300 K, which indicates that the thermal energy at low temperatures is insufficient to excite the oxygen ions^28^. At room temperature (300 K), the conduction mode is based on nearest neighbor hopping, which means that the electrons can obtain enough thermal energy to hop to the nearest trap. However, at low temperature (77 K), the conduction mode switches to variable range hopping. This means that the electrons with lower thermal energy can only seek for a hopping pathway with a lower energy barrier. This causes the overall path length to increase. Such a description can explain the high insulating region under the operation at 77 K. Moreover, this also confirms the semiconducting behavior of the filament. The activation energy in SnO_*x*_ is extracted as 11 meV based on temperature measurement (Supplementary Fig. 8). A control sample using Pt as top electrode also shows double set and reset behavior, which can exclude the presence of a metallic filament (Supplementary Fig. 9). Another control sample shows no difference between SnO_*x*_/Cr/Au and SnO_*x*_/Au devices, which indicates that the Cr layer cannot influence the switching behaviors (Supplementary Fig. 10).

### The mechanisms and simulation of SnO_*x*_/SnSe/SnO_*x*_ heterostructure

**Fig. 3:**
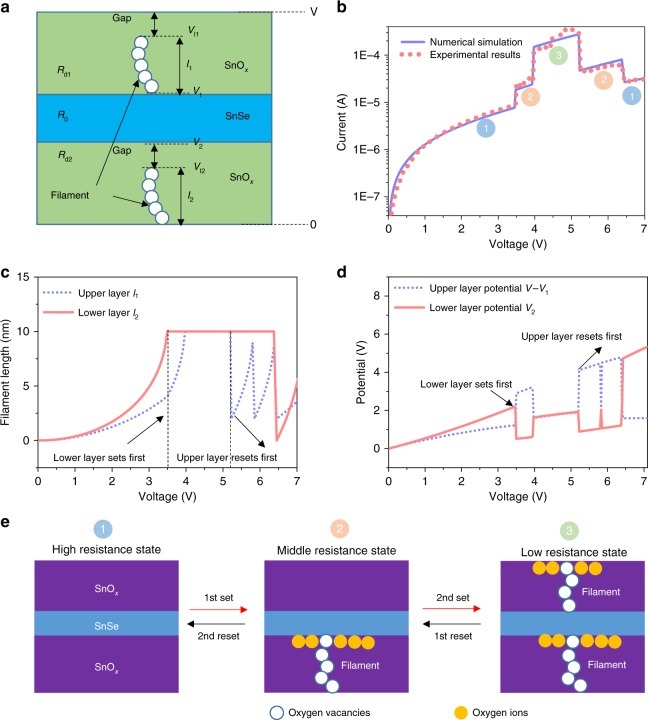
Theoretical model and simulations for SnSe RRAM. **a** A theoretical model for the SnO_*x*_/SnSe/SnO_*x*_ heterostructure. **b** The simulated switching behavior showing good fit with experimental results for the two-step set and reset behavior. **c** The filament length vs. the voltage in the SnO_*x*_/SnSe/SnO_*x*_ heterostructure. **d** The filament potential vs. the voltage in the SnO_*x*_/SnSe/SnO_*x*_ heterostructure. **e** Proposed filament growth and rupture mechanism to explain the double set and reset processes

The set and reset mechanisms are different in unipolar devices. For the set process, the electrical field can promote the growth of the filament. There is a positive feedback process during set whereby the electrical field promotes the growth of the filament, which results in a more conductive RRAM with larger Joule heating. This in turn improves the growth of the filament and hence completes the feedback loop. For the reset process, the Joule heating can break the filament. There is a competition between the filament rupture and filament growth during the reset process. At a higher voltage, more Joule heating can break the filament, while the filament growth rate also increases. These mechanisms were verified by reports of previous unipolar RRAM^27,29^. A theoretical model is established by combing SCLC theory, tunneling effect and Joule heating effect (Fig. 3a). (More details can be found in the Methods section and Supplementary Discussion). This model is used to fit the experimental double set/reset curve to further interpret the inner mechanism of the proposed device. Following this exercise, the whole double set and reset process can be understood deeply based on this model (see Methods for details). As shown in Fig. 3b, our simulation results fit well with the experimental results, which indicates that the model is suitable for SnO_*x*_/SnSe/SnO_*x*_ heterostructure. In this way, the double set and reset process can be understood by analyzing the filament growth and rupture process. For double set process, since the filament growing speed for upper and lower SnO_*x*_ could not always be the same, the upper and lower layer of SnO_*x*_ can be set separately. As a result, double set can happen. Figure 3c shows the simulation of filament growth process with double set, which exhibits the different growing speed of the filament during the set process. In the reset process, as the filament for the upper and lower layer cannot be always the same, the Joule heating in upper and lower layer can be different. As a result, either upper or lower layer with more Joule heating can break first. Figure 3c shows the filament length during the voltage sweep. It shows that the filament in the bottom layer grows to 10 nm first at 3.5 V, followed by the upper layer filament reaching the top electrode at 4 V. In the reset process, the filament in the upper layer breaks first followed by the lower layer. Such sequence can be further explained by the potential distribution on the filament. As shown in Fig. 3d, before the first set, the lower layer has a high potential as compared to the upper layer. As a result, the lower layer sets first. Immediately after the lower layer sets, the potential in the lower layer drops and the potential in the upper layer increases sharply. This can lead to the set process happening in the upper layer. During the reset process, the filament in the gap of the upper layer breaks first as a result of the higher applied potential *V*_*l*1_. The whole double set and reset processes are shown in Fig. 3e, and these processes are matched with the switching behavior in Fig. 3b. Our model can also simulate the double set and reset behaviors at 77 K (Supplementary Fig. 11), which is a good proof of the correctness of our model.

### Markov chain properties of SnO_*x*_/SnSe/SnO_*x*_ heterostructure

**Fig. 4:**
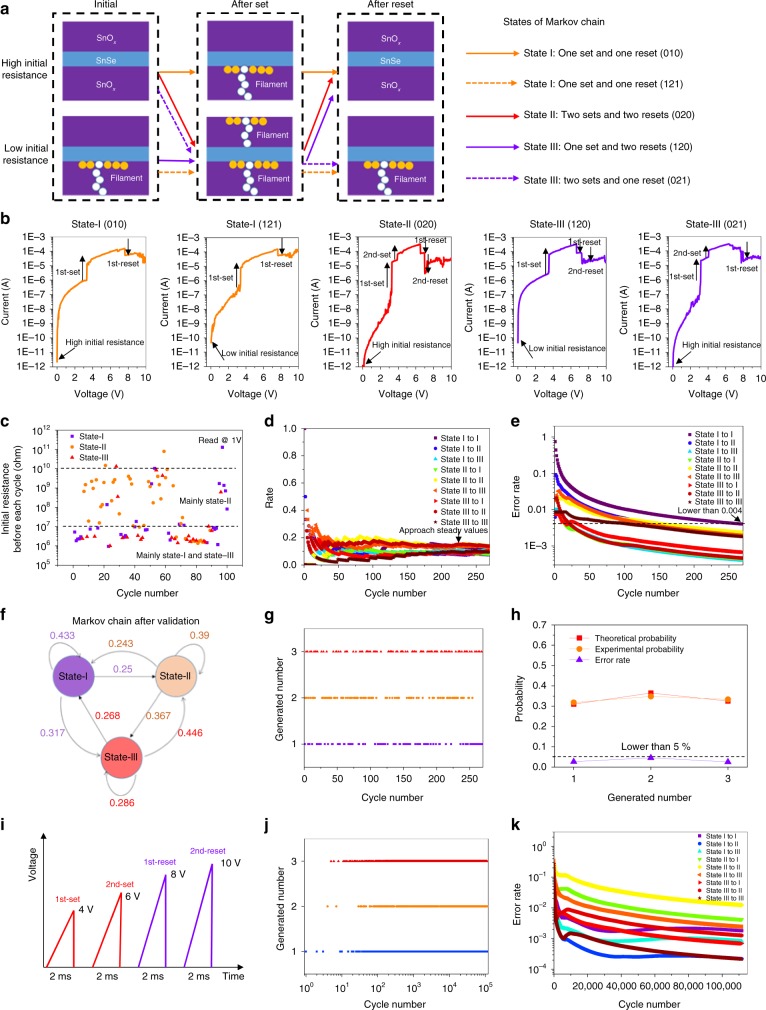
Demo of a single SnO_*x*_/SnSe/SnO_*x*_ device as one core of Markov chain. **a** The schematic showing the definition of three Markov states and these behaviors are quite related to the initial resistance state. There are five possible filament states insides the SnSe-RRAM: (i) 010, (ii) 121, (iii) 020, (iv) 120, and (v) 021 (numbers represents the conducting filament numbers in upper or lower layers. The first, second, and third numbers represents initial, after set and after reset states, respectively). In order to reduce the complexity of peripheral designs, the five filament states have been classified into three states of Markov chain. **b** The typical resistive switching behavior for state I: one set and one reset (010), state I: one set and one reset (121), state II: two sets and two resets (020), state III: one set and two resets (120), and state III: two sets and one reset (021). **c** The relation between initial resistance and the states in 100 cycles. **d** The probability for each state in 270 testing steps. **e** The error rate between each state showing continuous decrease after more testing steps. **f** The Markov chain after validation. The circular with certain stage in it represents the current or next stage. The arrow line between two state represents the state transfer direction and the number near it is the state transition probability between these two states (*p*_*ji*_), which is the conditioning probability of next state equal to *j* when the current state is *i* [*P*(*X*_*n*+1_ = *j*|*X*_*n*_ = *i*)]. **g** Demonstrating the usage of a Markov chain as fixed-probability random number generator. **h** Validation of the random number generator with error rate lower than 5%. **i** The four sawtooth waves as one cycle input signal to measure the device with faster speed and lower power. **j** The generated numbers in ~1.1 × 10^5^ cycles operation of SnSe-RRAM. **k** The error rate vs. cycle number

The *I–V* curves indicates five major kinds of filament states (Fig. 4a) of the devices: (i) 010, (ii) 121, (iii) 020, (iv) 120, and (v) 021. The number represent the conducting filament numbers in upper or lower layers. The first, second, and third numbers represent initial, after set and after reset states, respectively. In order to reduce the complexity of peripheral designs (see details in Supplementary Fig. 12), the five filament states have been classified into three states of Markov chain: state-I [one set and one reset (010), one set and one reset (121)], state-II [two sets and two resets (020)] and state-III [one set and two resets (120), two sets and one reset (021)]. In our device, we have two layers of the metal–oxide film. The rupture of the filament in the first layer of the metal–oxide film can always happen. However, in the second layer of the metal–oxide film, it can result in less Joule heating as a consequence of the rupture of the first filament with larger resistance. Moreover, a higher voltage can improve the competition between the filament rupture and the filament growth. So the filament breakage of the second layer of metal–oxide film is much harder than the first layer. Figure 3c shows the simulation results of filament growth and rupture process. The two sets can happen directly due to the positive feedback of the filament formation. However, for the reset process, after the first reset, the broken filament regrows twice, which causes the overall reset process to be slower and is also a proof that there is significant competition between the filament rupture and filament growth during the reset. This is the reason why in some cases, only one reset happens, which can result in the occurrence of state-I or state-III. The typical resistive switching curve for state I, state II, and state III are shown in Fig. 4b. During the classification, only resistance change larger than two times can be regarded as an effective set/reset. During the experiment, we found that the transfer between states shows Markov chain property, which means that the probability of the next state happening only depends on the current state and is irrelevant to all of the past states. As shown in Supplementary Fig. 13a, for conventional RRAM, the gap distance is only influenced by the previous reset. However, the residual filament is influenced by all the switching history. As shown in Supplementary Fig. 13b, the switching state (one set or two sets) belonging to state-I, state-II, or state-III is only influenced by the previous reset conditions. As shown in Fig. 4c, the high-initial resistance (before each cycle) in the range of 10^7^–10^10^ ohm can result in state-II happening with a higher probability, while state-I or state-III mainly distributes with lower initial resistance (before each cycle) below 10^7^ ohm. We also noticed that partial state-I or state-III distributes with high-initial resistance, which is in agreement with the two filament structures in state-I and state-III proposed in Fig. 4a. Figure 4c indicates that the three states are quite related to the initial resistance. The states can be also justified by reading the initial states of the RRAM. The Markov property can be described as follows:

1 $$\begin{array}{l}P\left( {X_{n + 1} = j|X_n = i,X_{n - 1} = i_1,X_{n - 2} = i_2,...,X_0 = i_n} \right)\\ = P\left( {X_{n + 1} = j|X_n = i} \right).\end{array}$$

In order to vertify Eq.(1), we investigate the frequency distribution of nine state-transfer conditions (*I* → *I*, *I* → *II*, *I* → *III*, *II* → *I*, *II* → *II*, *II* → *III*, *III* → *I*, *III* → *II*, *III* → *III*) by Eq. (2)

2 $$\hat p_{ji}^m = \frac{{\mathop {\sum}\limits_{k = 1}^m {I\left( {i \rightarrow j|i_0,j_0} \right)} }}{{\mathop {\sum}\limits_{k = 1}^m {I\left( {i|i_0} \right)} }}{.}$$

where $\hat p_{ji}^m$ is frequency distribution from state *i* to state *j* during the first *m* cycles, and *I*(*x|x*_0_) is the decision function whose definition can be written as:

3 $$I\left( {x|x_0} \right) = \left\{ {\begin{array}{*{20}{c}} 1, \\ 0,\end{array}} \right.\begin{array}{*{20}{c}} {x = x_0}; \\ {x \ne x_0}. \end{array}{}$$

With the testing cycles increasing, we found frequency distribution of all transfer states approaches steady values (Fig. 4d), indicating that under a given transfer state, Eq. (2) has a limit at infinite testing cycles and this limit value is equal to transfer probability according to the law of large numbers.

4 $$p_{ji} = \mathop {{\lim }}\limits_{m \to \infty } \hat p_{ji}^m{.}$$

In order to show the total mismatch between frequency distribution of transfer states, the mismatch rate is introduced (Eq. (5)). The result is as shown in Fig. 4e—as the cycle number increases, the mismatch rate of all state transfer conditions decreases quickly. Further, after 270 testing cycles, this rate can be lower than 10^−3^, which proves that the Markov property matches well with our proposed single device (Fig. 4f).

5 $$E_{ji}^m = \frac{1}{m}\mathop {\sum}\limits_m {\left( {p_{ji} - \hat p_{ji}^m} \right)} ^2{.}$$

For developing / testing a practical application involving the Markov property of the device, we demo a fixed probability random number generator such that a certain state appears randomly in a large cycle number scale. Fixed-probability random numbers have vital applications in big data processing and optimization such as simulated annealing algorithm^30^, genetic algorithm^31^, and Monte-Carlo simulations^32^. However, this type of random number generated by software is usually a pseudo-random number achieved by shift registers and XOR gate^33^, which shows large periodicity and is not a true random number. As to our random number generator, it depends on the Markov property, therefore periodicity does not appear in this device. Previous reported operation mechanism of 0/1 RNG application are mainly based on random telegraph noise^34^ or set voltage variability^35^. For our device, RNG application is realized based on Markov chain algorithm with three values per bit. Moreover, if the bits are increasing, three numbers per bit in our devices array configuration can enable an even larger amount of random numbers compared to the two numbers “0/1” per bit in conventional RRAM devices. The states-I, -II, and -III represent random numbers 1, 2, and 3. As shown in Fig. 4g, the generated number shows random distribution.

According to the state transition diagram of our implemented Markov chain, we can obtain the transition probability matrix *A*, which can be written as

6 $$A = \left[ {\begin{array}{*{20}{c}} {0.433} & {0.250} & {0.317} \\ {0.243} & {0.390} & {0.367} \\ {0.268} & {0.446} & {0.286} \end{array}} \right]{.}$$

If we define the probability of state I, II, and III as *π*_1_, *π*_2_, *π*_3_, and the initial state is state I which is extracted from experimental results, according to the Markov probability theory, the equation group (7) must be satisfied:

7 $$\left\{ {\begin{array}{*{20}{c}} {\pi _1 + \pi _2 + \pi _3 = 1}; \\ {[\pi _1\,\pi _2\,\pi _3]A = [\pi _1\,\pi _2\,\pi _3]}. \end{array}} \right.$$

By solving this equation group, we can obtain three theoretical probabilities separately. In our case, the result is 0.3102, 0.3648, and 0.3250 (More details are described in Supplementary Discussion). Meanwhile, after 270 testing cycles, the frequency distribution of the three states is 0.3186, 0.3481, 0.3333, respectively. The error rate of the three states are all below 5% (Fig. 4h), which indicates that our hardware fixed-probability random number generator is highly consistent with theoretical design and can be put into practical applications. Moreover, five devices are analyzed with similar probability distribution (Supplementary Table 1). All the devices show the relation of P (state II) > P (state-III) > P (state-I), and the total discrepancy to the theoretical values are all below 4%, which prove the good uniformity of the sequence by different devices.

In order to measure the device in a faster manner, the test method based on sawtooth waves has been developed (Fig. 4i), which is similar to the concept of “pulse” operation. When compared with pulse operation, sawtooth waves are similar to the DC sweep. In such an operation manner, SnSe RRAM has good durability up to 1.1 × 10^5^ cycles. By analyzing the total times of set and reset, the state-I, -II, and –III can be determined and random numbers can be obtained (Fig. 4j). Moreover, the error rate between each state shows continuous decrease after more testing cycles (Fig. 4k), which validates the Markov chain algorithm inside the SnSe-RRAM. For DC sweep, it will take 3 s for one sweep and the average power consumption for 270 DC sweeps is 3.75 × 10^−4^ W. For the sawtooth waves operation, the average power consumption is 0.9 × 10^−7^ W during 1.1 × 10^5^ cycles. The random numbers generated by our SnSe RRAM were also checked by NIST test (see details in Supplementary Discussion). The final NIST report reveals that the random numbers generated by our devices have overall good randomness and the potential to be applied in industry in the future.

In order to highlight the advance of using a single device as a fixed-probability random number generator, we also compare the hardware resources required for implementing a software approach with the same function—and the requirement is around 13,700 transistors! (see Supplementary Fig. 14). Therefore, a Markov chain (core) realized via a single device can simplify the system enormously, and open new application areas in data optimization and machine learning. For large scale integration and for making multiple Markov cores, wafer-scale SnSe films can be grown by the chemical vapor deposition method.

## Discussion

In summary, a Markov chain algorithm is successfully built in a single RRAM device where the resistive switching layer is based on a multilayer SnSe material whose both sides develop native oxide with atomic flatness. A unique two-step set and reset behavior is observed. Numerical simulation can reproduce such unique behavior and provide a deeper understanding of the switching sequence. The three states of a Markov chain are defined based on the step number of the set and reset. Moreover, we demonstrate that the three states are only related to the current states instead of the overall history of the system, which validates the Markov chain. A fixed-probability random number generator is also demonstrated for practical applications.

## Methods

### Device fabrication

Bottom electrode line with 500 nm width was first patterned by e-beam lithography, followed by thermal evaporation of Cr/Au with 3/35 nm thickness. Then SnSe crystal was exfoliated on the bottom metal line by the well-known mechanical exfoliation method^36^. After the SnSe flakes were identified by optical microscopy, another e-beam lithography followed by thermal evaporation of Cr/Au with 3/35 nm thickness and lift off was performed to fabricate the top electrodes (also 500 nm wide). In this way, a cross-bar structure (with bottom electrode) was formed. For SnSe-RRAM, the oxidation condition can be described as the oxidation at room temperature (300 K), in air (21% oxygen) for 24 h.

### XPS characterization

The XPS spectra was collected using monochromatic 1486.7 eV Al Ka X-ray source on PHI VersaProbe II X-ray Photoelectron Spectrometer with a 0.47 eV system resolution. The energy scale had been calibrated using Cu 2p3/2 (932.67 eV) and Au 4f7/2 (84.00 eV) peaks on a clean copper plate and a clean gold foil.

### AFM characterization

The AFM images were captured using a Bruker Dimension-Icon FastScan system.

### SEM and EDX characterization

The micrographs were taken using the Hitachi SU8230 CFE SEM with EDX function.

### Electrical measurement

The electrical measurement was conducted by two-point probing the top and bottom electrodes of the device in a Lakeshore cryogenic probe station with the pressure well below 10^−4^ T. Such measurement condition can exclude the influence from water and moisture, which can reveal the intrinsic behaviors of the SnSe-RRAM. Temperature dependent measurements down to 77 K were performed in the Lakeshore probe station with substrate cooling via liquid nitrogen. DC single sweep from 0 to 10 V was measured by B1500A, and up to 270 such cycles were applied. The sawtooth waves were generated by B1500A with B1542A pulse IV package.

### Numerical modeling the resistive switching in SnO_*x*_/SnSe/SnO_*x*_ heterostructures

The theoretical model is shown in Fig. 3a where the interlayer SnSe can be regarded as series resistance without change during the switching. The initial filament length after forming are *l*_1_ and *l*_2_, respectively. There is a gap region between the filament and top electrode both for the top and bottom SnO_*x*_ layers. The gap is assumed to be physically located at the top of each filament. The set and reset process can be described by bridging or breaking the gap respectively. *V*_*l*1_ and *V*_*l*2_ represent the potential on the top of the filament for upper and lower SnO_*x*_ layers, respectively. *V*_1_ and *V*_2_ represent the top and bottom potential of SnSe. Since the current flow should pass through the boundaries of the upper gap region of SnO_*x*_, upper filament region of SnO_*x*_, interlayer SnSe, lower gap region of SnO_*x*_*,* and the lower filament region of SnO_*x*_, therefore the equation set can be written as:

8 $$\left\{ {\begin{array}{*{20}{c}} {\begin{array}{*{20}{c}} {I = \frac{{V - V_{l_1}}}{{R_{d1}}} + f\left( {V - V_{l_1},T_0 + aI\left( {V - V_{l_1}} \right),l_1} \right)}; \\ {I = \frac{{V_{l_1} - V_1}}{{R_{l1}}}}; \end{array}} \\ {\begin{array}{*{20}{c}} {I = \frac{{V_1 - V_2}}{{R_0}}}; \\ {I = \frac{{V_2 - V_{l_2}}}{{R_{d2}}} + f\left( {V_2 - V_{l_2},T_0 + aI\left( {V_2 - V_{l_2}} \right),l_2} \right)}; \\ {I = \frac{{V_{l_2}}}{{R_{l2}}}.} \end{array}}{} \end{array}} \right.$$

where *V* is the electric potential between tunneling distance, *T* is the temperature, *l* is the length of CF; *R*_0_ is the resistance of SnSe. For simplification, the function *f* (*V*, *T*, 1) represents the tunneling current, which can expressed as:

9a $$I = Aq\frac{{8\pi ^2m}}{{h_0^3}}\frac{{kT}}{{c_1\sin \left( {\pi c_1kT} \right)}},\hfill \hfill V < \phi _0{.}$$

9b $$I = A\frac{{4\pi q^2m}}{{h_0^3\alpha ^2\phi _0}}\left( {\frac{V}{{h - l}}} \right)^2e^{ - \frac{{2\alpha \left( {h - l} \right)\sqrt{q\phi _0^{3}}}}{{3V}}},\hfill\hfill\ V > \phi _0{.}$$

where *A* is the area of filament, *m* is effective electron mass, *h*_0_ is Planck’s constant, and *ϕ*_0_ is the barrier height with zero applied bias. The parameter *c*_1_ satisfies Eq. (10)

10 $${\it{c}}_{\mathrm{1}} = \left\{ {\begin{array}{*{20}{l}} {\frac{{\alpha \left( {h - l} \right)}}{{V\sqrt q }}\left( {\phi _0^{1/2} - \left( {\phi _0 - V} \right)^{1/2}} \right)} {,}\hfill & {{}\,V < \phi _0}; \hfill \\ {\frac{{\alpha \left( {h - l} \right)}}{{V\sqrt q }}\phi _0^{1/2}} {,}\hfill & {{}\,V > \phi _0} .\hfill \end{array}}\right.{}$$

and the parameter *α* satisfies Eq. (11)

11 $$\alpha = \frac{{2\sqrt {2m} }}{\hbar }{.}$$

Ion drift causes the device to experience localized heating and has an effect on the *I*–*V* property. We simplify the Joule heating as a linear process, which can be written as Eq. (12)

12 $$T = T_0 + aIV.$$

Using Eq. (13) to estimate the CF length at a certain time,

13 $$\begin{array}{l}l_1\left( {t_0 + \Delta t} \right) = l_1\left( {t_0} \right) + \frac{{dl_1}}{{dt}}\Delta t;\\ l_2\left( {t_0 + \Delta t} \right) = l_2\left( {t_0} \right) + \frac{{dl_2}}{{dt}}\Delta t.\end{array}{}$$

the growth rate of CF is written as:

14a $$\frac{{dl}}{{dt}} = 2dv\exp \left( { - \frac{{qU_a}}{{kT}}} \right){\mathrm{sinh}}\left( {\frac{{qVd}}{{2kT\left( {h - l} \right)}}} \right),\hfill \hfill U_a - \frac{{qV}}{{2\left( {h - l} \right)}} > 0{.}$$

14b $$\frac{{dl}}{{dt}} = s\exp \left( { - \frac{{qU_a}}{{kT}}} \right){\mathrm{sinh}}\left( {\frac{V}{{V_0}}} \right), \hfill \hfill {}\,U_a - \frac{{qV}}{{2\left( {h - l} \right)}} < 0{.}$$

## Electronic supplementary material

Supplementary Information

## Data Availability

The data that support the results of this study are available from the corresponding author on reasonable request. See author contributions for specific data sets.
