# Supplementary material for: A hardware Markov chain algorithm realized in a single device for machine learning
Source: Nat Commun. 2018 Oct 17;9:4305. doi: 10.1038/s41467-018-06644-w (PMC6192990; doi:10.1038/s41467-018-06644-w)
Supplement: Supplementary file 1 — Supplementary Information [file 41467_2018_6644_MOESM1_ESM.pdf]

Supplementary Information

A hardware Markov chain algorithm realized in a single device for machine learning

He Tian[1, 2, †,*], Xuefeng Wang[1, 2, †], Mohammad Ali Mohammad[3], Guang-Yang Gou[1, 2], Fan Wu[1, 2], Yi Yang[1, 2], Tian-Ling Ren[1, 2, *]

[1]*Institute of Microelectronics, Tsinghua University, Beijing 100084, China*

[2]*Beijing National Research Center for Information Science and Technology (BNRist), Tsinghua University, Beijing 100084, China*

[3]*School of Chemical and Materials Engineering (SCME), National University of Sciences and Technology (NUST), Sector H-12, Islamabad 44000, Pakistan*

[†] These authors contributed equally to this work

*Corresponding author. Email: tianhe88@tsinghua.edu.cn (H.T.); RenTL@tsinghua.edu.cn (T.-L.R.)

Supplementary Figures

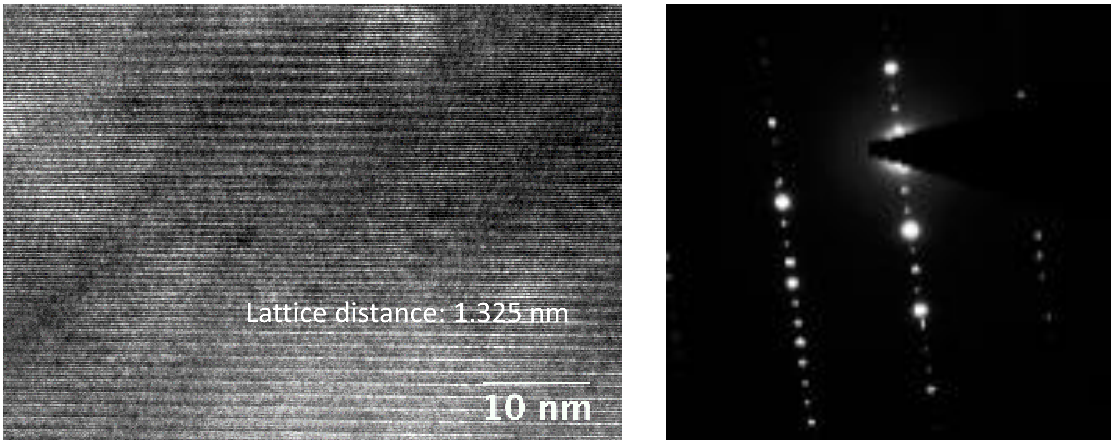

Supplementary Figure 1 | A transmission electron microscope (TEM) of SnSe. The SnSe sample (~200 nm wide) is observed by transmission electron microscopy (TEM) under a 200 kV beam. An interlayer distance of 1.325 nm is observed and the diffraction image indicates a high quality crystal structure.

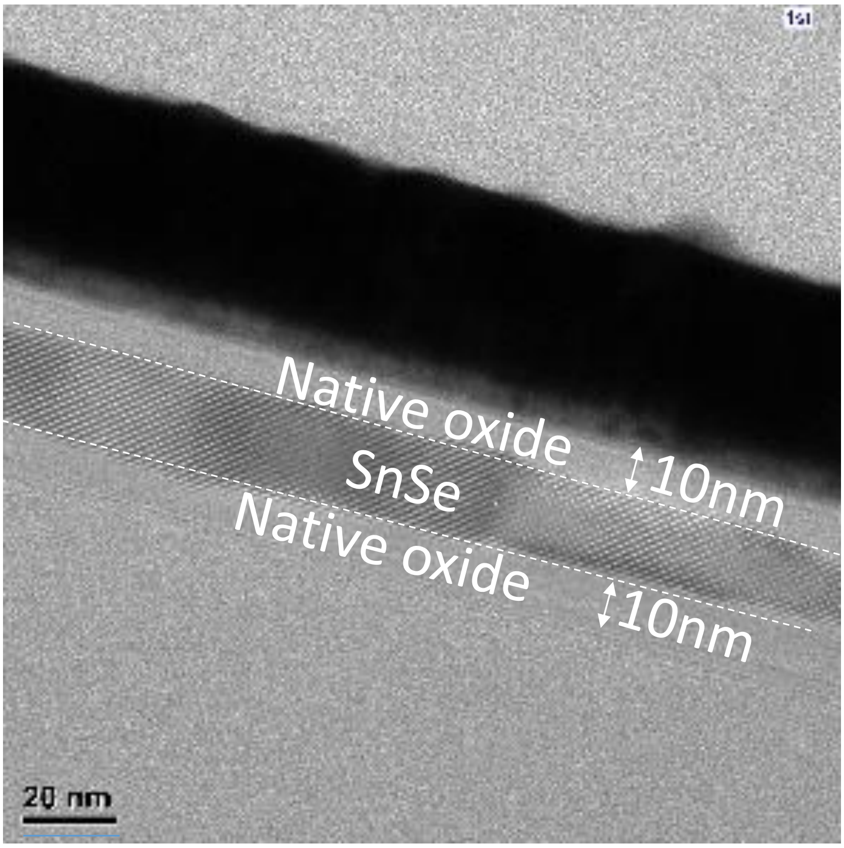

Supplementary Figure 2 | A TEM cross-section of an oxidized SnSe flake. In order to investigate the oxidation process, a cross-section of another 40 nm wide oxidized SnSe sample is observed under TEM with 250 kV voltage. The SnSe crystal shows two 10 nm amorphous layers sandwiching the interlayer SnSe. TEM cross-section was prepared by the exfoliation of the SnSe flake on a SiO_2/Si substrate followed by Pt capping and focused ion beam (FIB) milling.

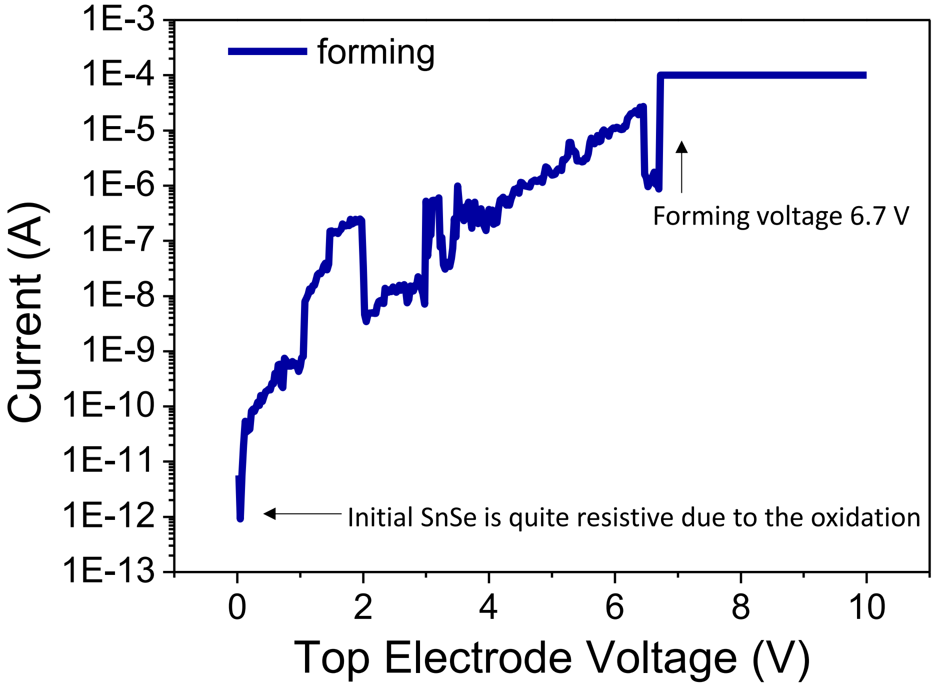

Supplementary Figure 3 | A forming curve for SnO_x/SnSe/SnO_x heterostructure.

The forming voltage is 6.7 V.

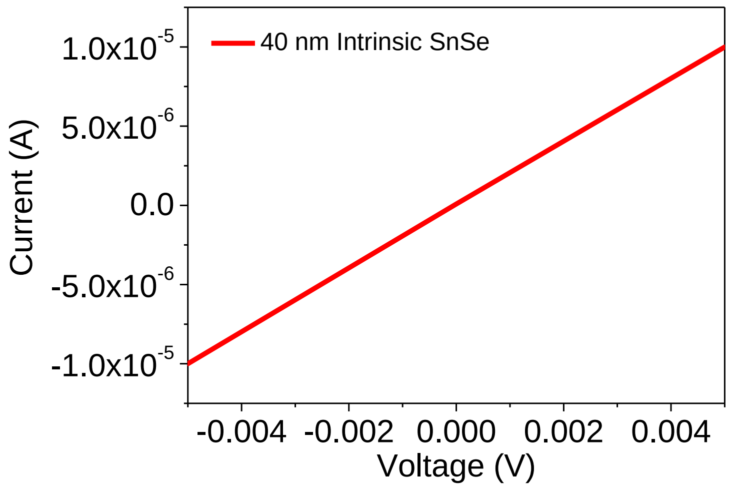

Supplementary Figure 4 | *I-V* characteristics for vertical conductivity of pristine SnSe.

Very good *I-V* characteristics are shown with just 500 Ω resistance without resistive switching effects.

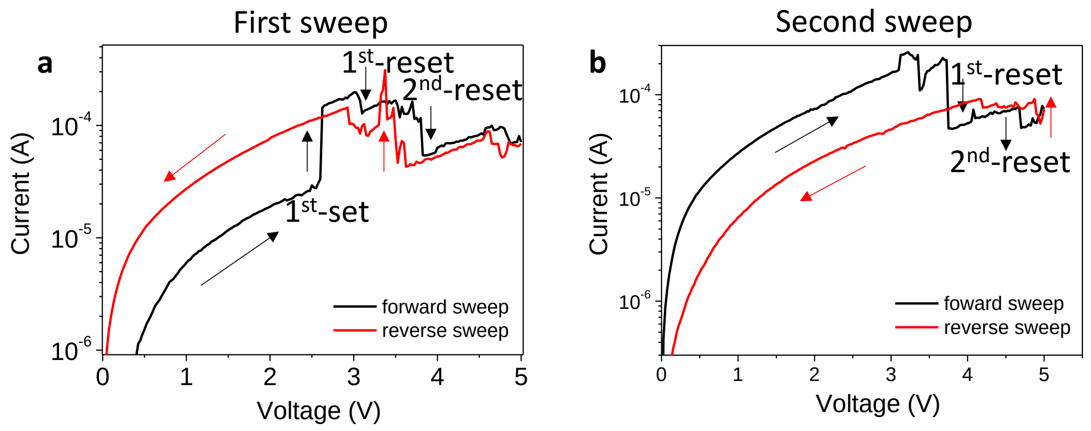

Supplementary Figure 5 | Reverse sweep immediately after the forward sweep. (a) The first sweep; (b) The second sweep. For unipolar RRAM, the filament formation (set) is due to the E-field driving the oxygen ions to form a pathway of oxygen vacancies. And the filament rupture (reset) is due to the Joule heating breaking the filament. Moreover, in the reset process, there is a competition between the filament growth and the filament rupture process. In Supplementary Figure 5a, during the forward sweep, one set and two resets are observed, which belong to state-III. In the reverse sweep, the device sets again at 3.5 V, which indicates the competition mechanism also being present in the reset region. During the reverse sweep, since the device is already in the off-state, therefore the Joule heating is not obvious. The influence of E-field is dominant, which can cause a set to occur again in the reverse sweep. Supplementary Figure 5b shows another sweep. Since the device is initially in a low-resistance state, only two resets were observed in the forward sweep. In the reverse sweep, the set is observed again, which confirms the competition mechanism in the reset region.

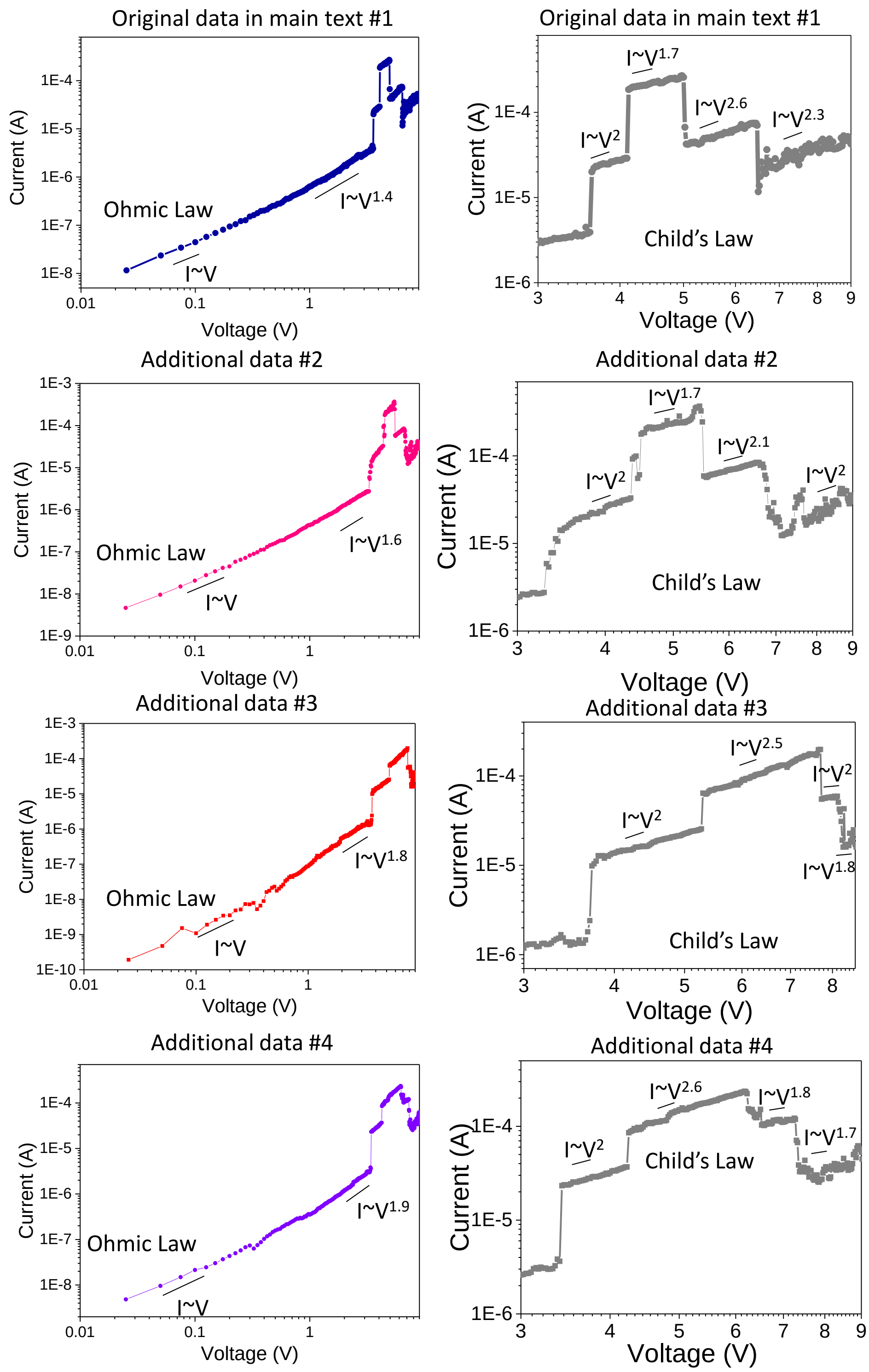

Supplementary Figure 6 | The fitting of resistive switching curves. The original data from the manuscript main text along with three more measurements demonstrating the Ohmic law at low voltage bias and Child's law at high voltage bias, which is a proof of SCLC theory.

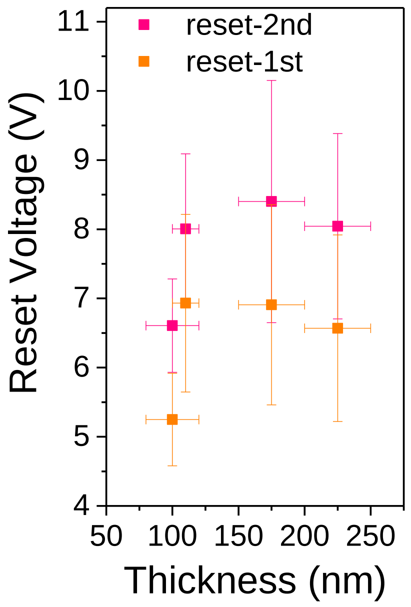

Supplementary Figure 7 | The reset voltage vs. film thickness. As compared with the set voltage vs. thickness plot in Fig. 2f, the reset voltage vs. thickness plot has larger voltage distributions. This is due to the dynamic and competing effects between filament growth and filament rupture during the reset process[1]. In general, the 1st-reset voltage is located around 7 V and the 2nd-reset voltage is located around 8 V. The 100 nm thick sample shows slightly smaller reset voltages, which could be explained by the thinner SnSe with a smaller resistance. The error bars in y-direction are calculated based on the set voltage in 100 cycles for each device. The error bars in x-direction are calculated based on the thickness variations from AFM results for each SnSe flake

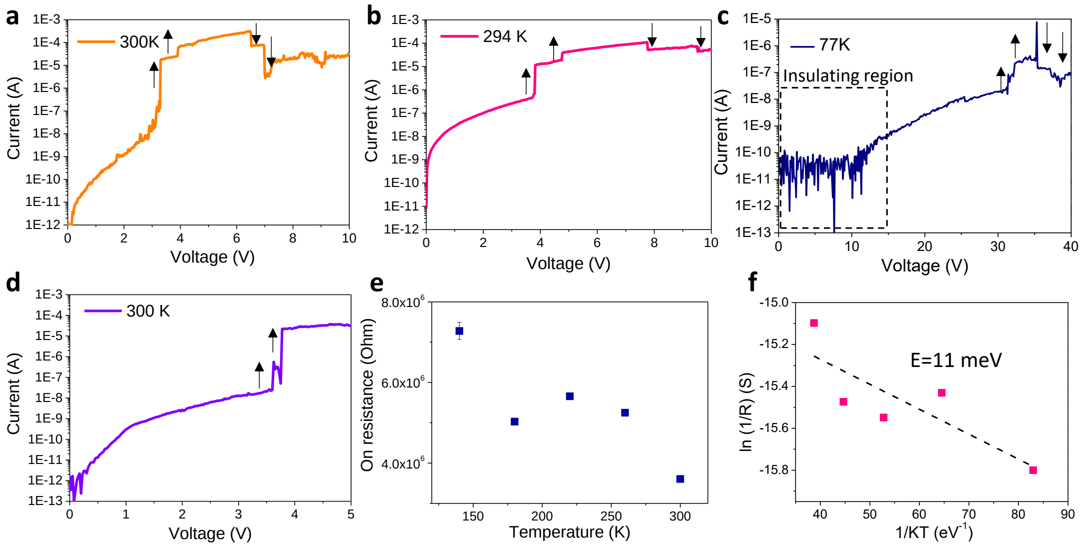

Supplementary Figure 8 | Temperature measurements of SnSe RRAM. The double set and reset behaviors at (a) 300 K, (b) 294 K and (c) 77 K along with a focus on (d) the double set process at 300 K. Also shown are (e) the on resistance vs. temperature after the double set at 300 K and (f) the ln(1/R) vs. 1/KT plot showing an activation energy of 11 meV based on the slope. As shown in panel (b), the double set and reset behaviors at 294 K shows slightly larger set and reset voltage than that of 300 K. To understand the filament type inside the SnO_x, temperature measurement is performed for a fully oxidized 25 nm SnO_x device. The device was pre-set to low resistance state ("on resistance") under room temperature (panel (d)) and then the "on-resistance" was measured under different temperatures. The error bars in panel (e) are calculated from the *I-V* measurements in two times. It shows that the on-resistance increases with decreasing temperature (panel (e)), which indicates a semiconducting filament instead of metallic filament. Further fitting of the slope shows the activation of oxygen ions energy around 11 meV (panel (f)), which is a reasonable value as compared to previous HfO_x-based RRAM with an activation energy from 4 meV to 40

meV. Such activation energy implies that the oxygen ions need 11 meV thermal energy to enable the jumping from one oxygen vacancy to another nearby vacancy.

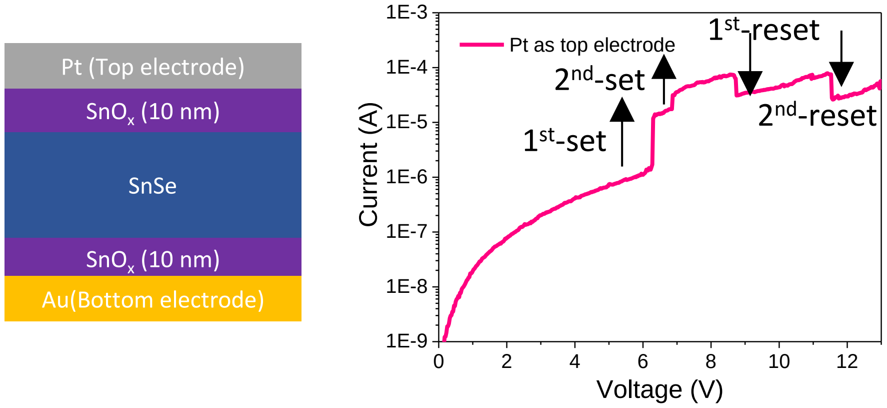

Supplementary Figure 9 | The control sample using Pt as top electrode. In order to fully exclude the influence of metallic filament from the top electrode on the double jump phenomenon, a control sample using Pt as top electrode is fabricated. Pt is always regarded as a noble electrode in oxide-based RRAM. As shown in Supplementary Fig. 9, the double set and reset behavior can also be reproduced in such a control sample. This is strong evidence that the double set and reset behavior is dominated by SnO_x/SnSe/SnO_x heterostructure instead of SnO_x/electrode interface. As the Pt is unable to preserve oxygen, the number of switching cycles are limited. Moreover, Pt has a higher work function, which results in Schottky emission and larger set and reset voltage. Although there were reports for the migration and nucleation of Pt filament, the required E-field is around 5 MV/cm. In our case, taking into account the set voltage around 6 V and SnO_x thickness of 10 nm on both sides with SnSe in the middle, the E-field can be lower than 3 MV/cm, which is insufficient to drive the Pt ions.

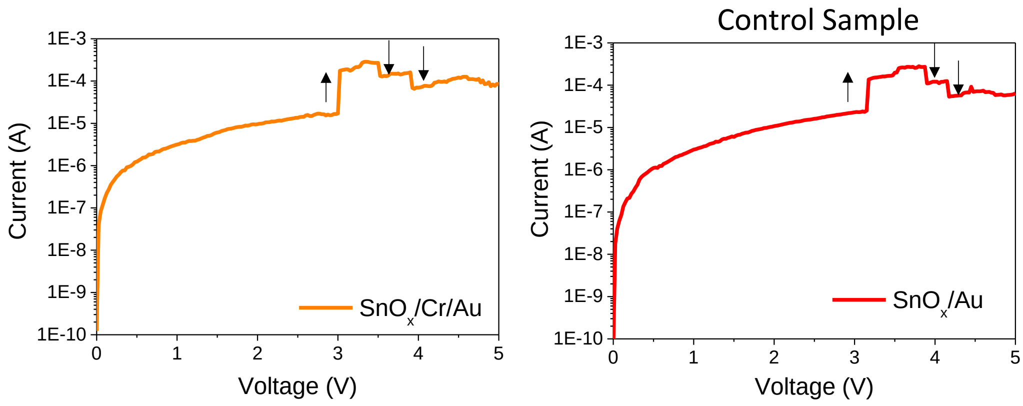

Supplementary Figure 10 | *I-V* performance for different top electrode metals. There is no considerable difference between the SnO_x/Cr/Au and SnO_x/Au devices. The Cr is only a 3nm thick adhesion layer and is quite conductive. Moreover, the switching mechanism is SCLC related filament change inside the SnO_x instead of Schottky emission at the interface between metal and oxide. Such 3 nm adhesion Cr layer cannot influence the switching behaviors.

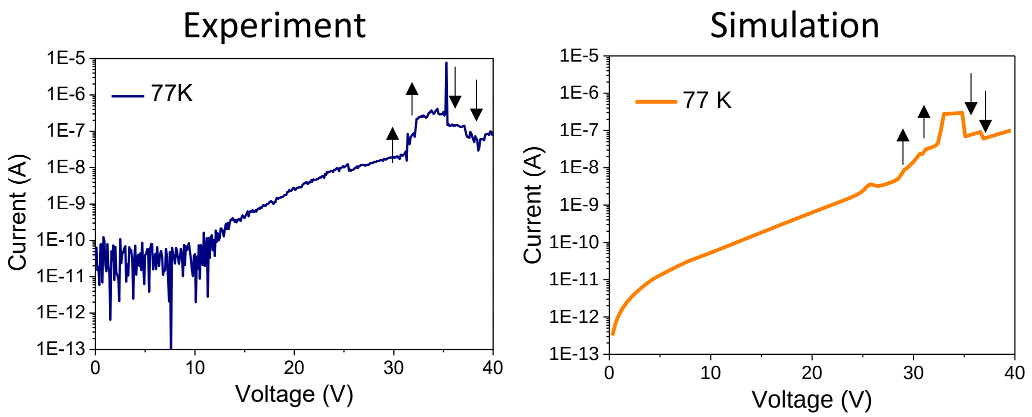

Supplementary Figure 11 | The experimental and simulation results at 77 K. Since the temperature (T) is also taken into account, the model can be also used at 77 K. The double set and reset behaviors can be reproduced at 77 K by simulations. The simulated values of set and reset voltage are quite similar to the experimental results, which is a good proof of the correctness of the model.

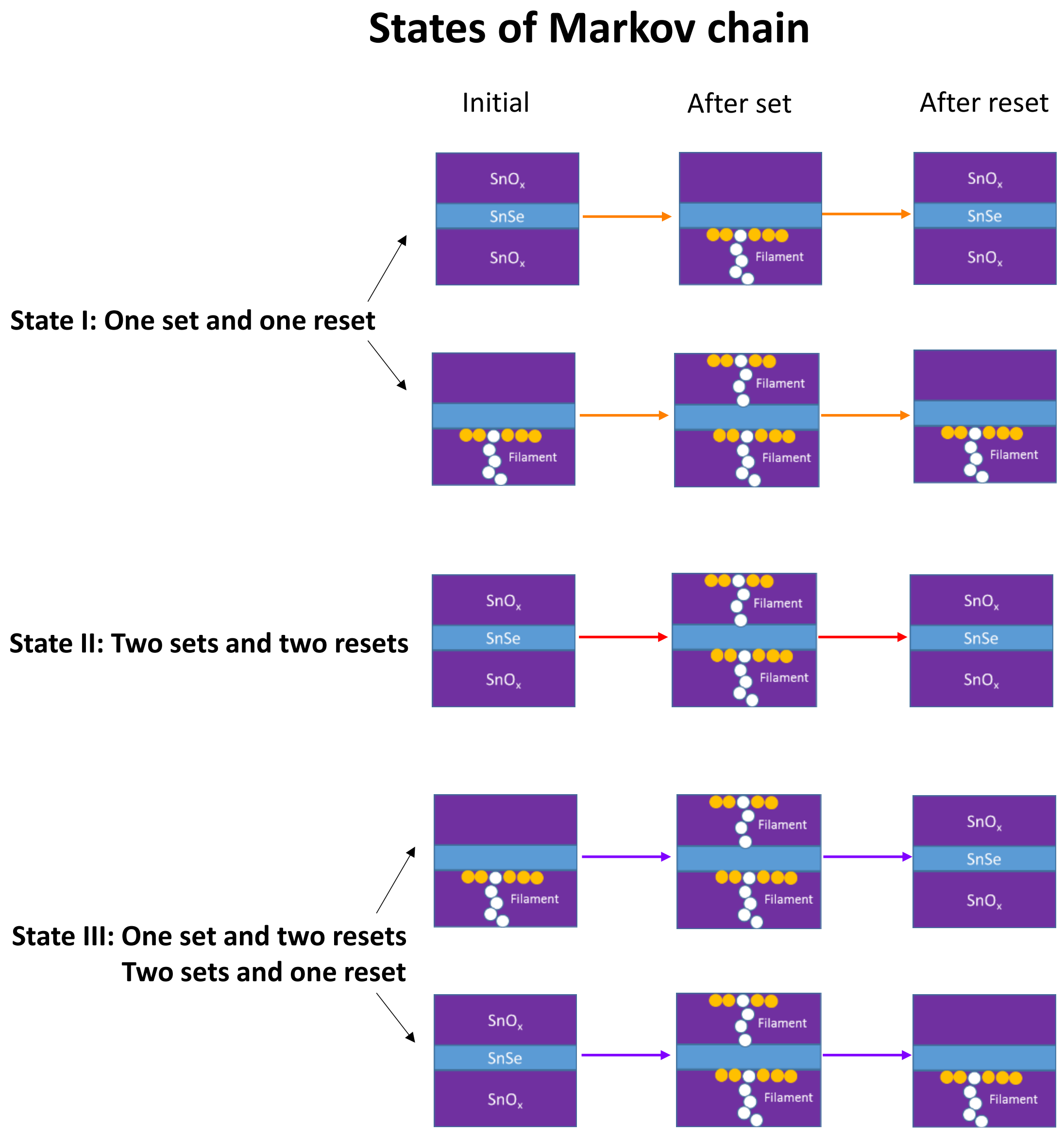

Supplementary Figure 12 | States of the Markov chain in SnSe-RRAM. The reason why we use three states is that three states can greatly reduce the complexity of peripheral designs. If three states (I, II, III) are utilized, the sum of the times required for abrupt jumps in current can distinguish them (state-I: jumping 2 times, state-II: jumping 4 times and state-III: jumping 3 times) and furthermore, the hardware implementation is relatively straightforward. But if five states are used, more shifters and comparators (in case of 010 and 121, as different judgment constants N are

needed), more logic judgment conditions (in case of 021 and 120, as set part and reset part are both needed to distinguish them) are required, which make the hardware resource consumption not optimized.

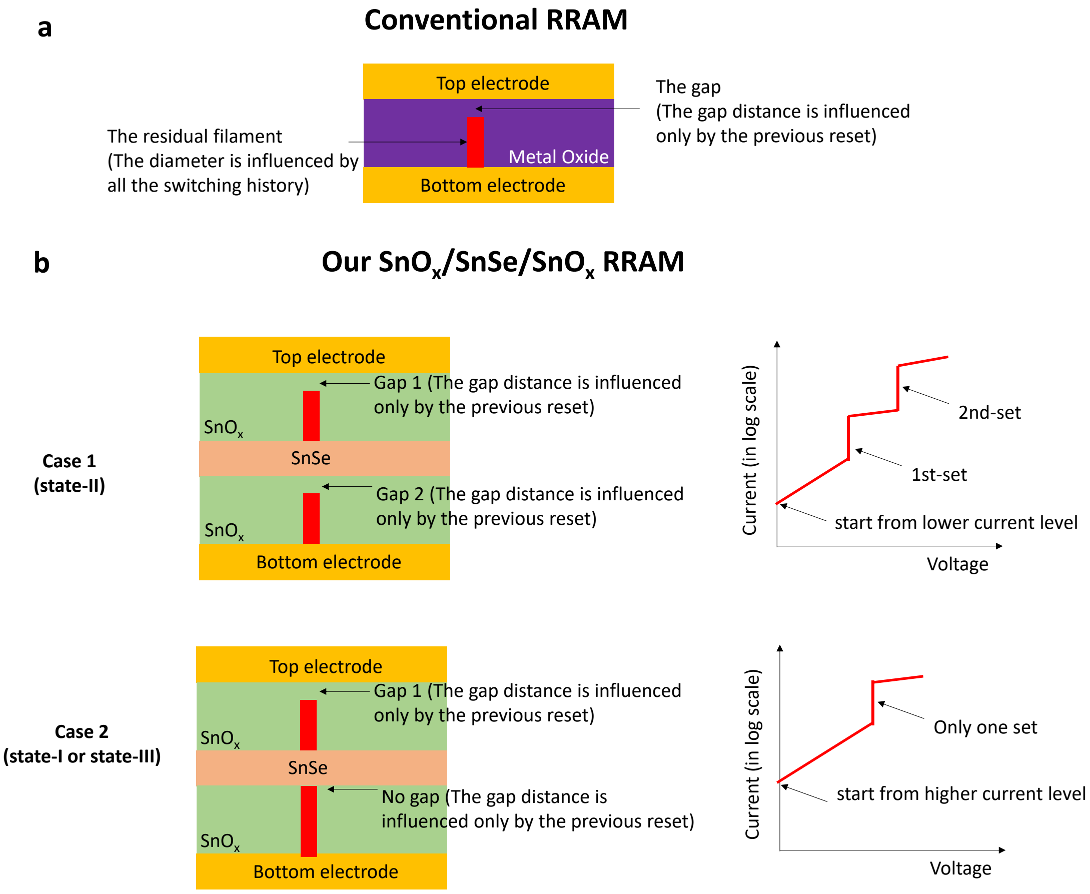

Supplementary Figure 13 | Operating mechanisms comparison. (a) The conventional RRAM mechanism. The gap distance is only influenced by the previous reset. The residual filament is influenced by the entire switching history. (b) Our SnO_x/SnSe/SnO_x RRAM mechanism. The switching state belonging to state-I, state-II or state-III is only influenced by the previous reset conditions.

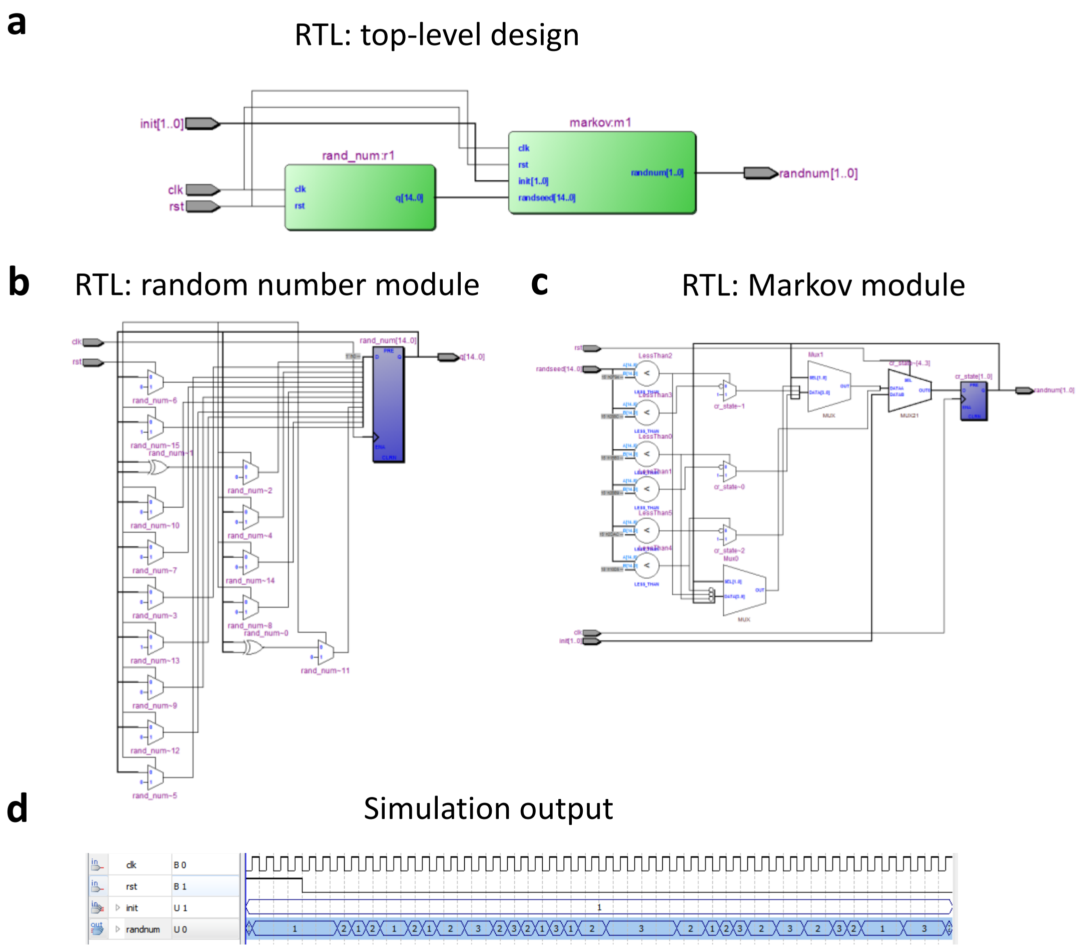

e Resource usage

Flow Summary	
Flow Status	Successful - Thu Apr 13 15:39:49 2017
Quartus II 64-Bit Version	13.0.1 Build 232 06/12/2013 SP 1 SJ Full Version
Revision Name	random
Top-level Entity Name	top
Family	Cyclone II
Device	EP2C35F672C8
Timing Models	Final
Total logic elements	50 / 33,216 (< 1 %)
Total combinational functions	49 / 33,216 (< 1 %)
Dedicated logic registers	16 / 33,216 (< 1 %)
Total registers	16
Total pins	6 / 475 (1 %)
Total virtual pins	0
Total memory bits	0 / 483,840 (0 %)
Embedded Multiplier 9-bit elements	0 / 70 (0 %)
Total PLLs	0 / 4 (0 %)

Total transistors: 50*274=13700

Supplementary Figure 14 | The software approach resource consumption. (a) The register transfer level (RTL) of the top-level design. (b) The RTL of the random number module. The role of this module is to initiate the software Markov chain

under a certain probability. The probability used in the software is the same as that extracted from the device (Fig. 4f) in order to make a fair comparison. The common pseudo-random number algorithm used here includes a 14-bit shift register and two exclusive-OR (XOR) operations. This is due to the fact that a 14-bit number can reach the minimum accuracy required for transfer probability and two XOR gates can guarantee the random property of a pseudo-random number. (c) The RTL of the Markov module. In order to achieve a Markov chain, we use finite-state machine (FSM) and define three fixed-probability random numbers as three states. For saving the register resource, we map the current state (cr_state) and next state (nx_state) to the same registers. As the sum of total transfer probabilities from given state to other states is 1, we can in order split sequence 0,1,2,…, 2^{14}-1 into 3 parts, the number of each part is equal to transfer probability multiplied by 2^{14}. Therefore the condition of state transfer can be simplified to judge which part the random number generated by random number module belongs to. (d) The simulation of fixed probability random number output. (e) The resource usage report. This report is generated after synthesizing and fitting the design to Altera Cycle II FPGA (EP2C35F672C8). From the report we can see that the design consumes 50 logic elements (LEs) and 16 registers. According to the datasheet of EP2C35F672C8, we find the structure of LEs and estimate the total transistor number of LE in a conservative way, which is about 274. As 16 registers are included in 50 LEs, we will exclude the resource consumption in this part. Therefore the total number of transistors required to achieve the proposed design is about 13700.

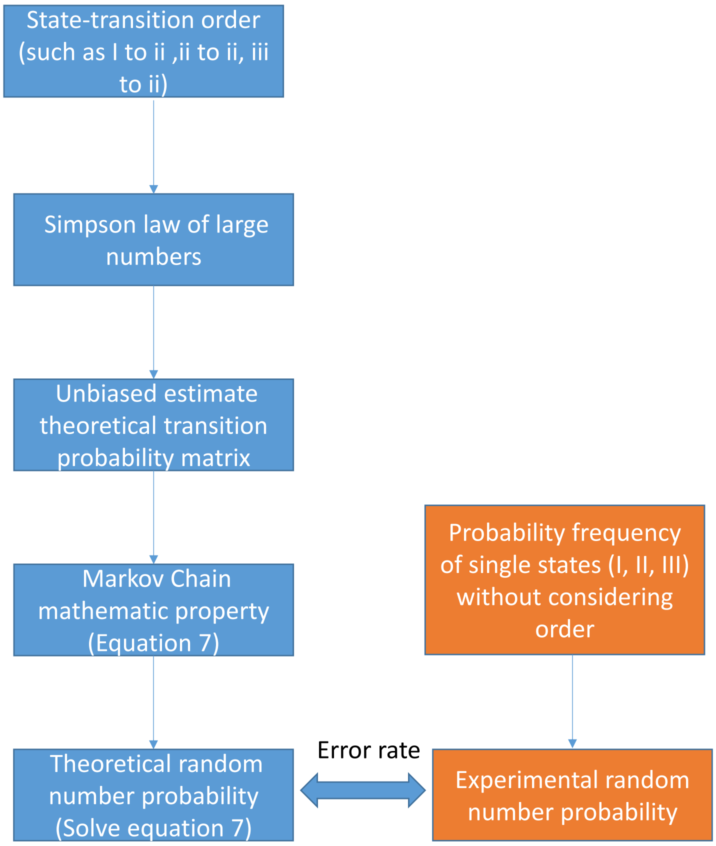

Supplementary Figure 15 | Calculation process for probability.

Supplementary Tables

Supplementary Table 1. The uniformity of the sequence generated by five devices.

Five devices are analyzed with similar probability distribution. All the devices show the relation of P (state II)>P (state-III)>P (state-I) and the total discrepancy when compared with the theoretical values are all below 4%, which prove the stable states.

	Probability of state I	Probability of state II	Probability of state III	Total discrepancy comparing to theoretical values
Original device in main text #1	0.3186	0.3481	0.3333	3.46%
Additional device #2	0.3085	0.3601	0.3314	1.38%
Additional device #3	0.3042	0.3790	0.3168	2.87%
Additional device #4	0.3070	0.3692	0.3238	0.94%
Additional device #5	0.3066	0.3709	0.3225	1.25%

Supplementary Table 2. Percentage probability of each state transfer.

State transfer	Percentage
i→i	0.1359
i→ii	0.0786
i→iii	0.0995
ii→i	0.0883
ii→ii	0.1415
ii→iii	0.1329
iii→i	0.0866
iii→ii	0.1441
iii→iii	0.0926
Total	1.0000

Supplementary Table 3. NIST testing results.

RESULTS FOR THE UNIFORMITY OF P-VALUES AND THE PROPORTION OF PASSING SEQUENCES

generator is <houchuli.txt>

C1	C2	C3	C4	C5	C6	C7	C8	C9	C10	P-VALUE	PROPORTION	STATISTICAL TEST
0	3	1	0	2	0	1	1	3	0	0.162606	11/11	Frequency
1	2	2	1	0	2	1	0	1	1	0.834308	10/11	BlockFrequency
1	2	0	2	2	1	2	0	1	0	0.637119	11/11	CumulativeSums
0	1	2	2	1	3	0	1	1	0	0.437274	11/11	CumulativeSums
2	0	3	0	2	0	3	0	0	1	0.090936	11/11	Runs
1	2	0	1	1	0	2	3	0	1	0.437274	11/11	LongestRun
1	3	1	0	2	1	0	1	1	1	0.637119	11/11	Rank
2	1	3	1	2	2	0	0	0	0	0.275709	11/11	FFT
3	3	0	0	1	1	1	0	0	2	0.162606	10/11	NonOverlappingTemplate
1	1	2	2	0	2	1	1	0	1	0.834308	11/11	NonOverlappingTemplate
0	1	0	2	2	0	2	2	1	1	0.637119	11/11	NonOverlappingTemplate
1	1	2	1	1	0	1	0	0	4	0.162606	11/11	NonOverlappingTemplate
0	0	1	2	1	2	2	1	0	2	0.637119	11/11	NonOverlappingTemplate
1	2	0	2	1	1	1	1	0	2	0.834308	11/11	NonOverlappingTemplate
0	2	2	1	0	3	1	1	1	0	0.437274	11/11	NonOverlappingTemplate
1	0	0	0	2	1	1	2	2	2	0.637119	11/11	NonOverlappingTemplate
0	1	0	2	0	2	3	2	1	0	0.275709	11/11	NonOverlappingTemplate
0	1	2	1	1	1	2	2	0	1	0.834308	11/11	NonOverlappingTemplate
1	0	1	2	1	2	1	0	1	2	0.834308	11/11	NonOverlappingTemplate
1	1	1	1	1	0	0	2	2	2	0.834308	11/11	NonOverlappingTemplate
0	3	0	1	0	0	2	1	2	2	0.275709	11/11	NonOverlappingTemplate
0	1	2	3	1	1	1	2	0	0	0.437274	11/11	NonOverlappingTemplate
1	2	1	1	0	1	1	2	0	2	0.834308	10/11	NonOverlappingTemplate
1	0	1	2	1	1	2	2	1	0	0.834308	11/11	NonOverlappingTemplate
0	1	0	2	2	1	3	1	0	1	0.437274	11/11	NonOverlappingTemplate
1	0	0	3	1	2	0	2	2	0	0.275709	11/11	NonOverlappingTemplate
1	0	0	1	1	2	1	0	1	4	0.162606	11/11	NonOverlappingTemplate
1	0	1	2	2	0	2	2	1	0	0.637119	10/11	NonOverlappingTemplate
2	0	1	3	2	0	1	1	0	1	0.437274	10/11	NonOverlappingTemplate
2	1	2	1	1	1	1	1	0	1	0.964295	10/11	NonOverlappingTemplate
0	1	1	4	0	0	1	2	1	1	0.162606	11/11	NonOverlappingTemplate
1	0	1	2	0	1	1	3	2	0	0.437274	11/11	NonOverlappingTemplate
2	1	2	2	0	2	1	1	0	0	0.637119	11/11	NonOverlappingTemplate
0	1	2	1	1	2	0	2	1	1	0.834308	11/11	NonOverlappingTemplate
1	2	2	1	0	1	2	0	0	2	0.637119	11/11	NonOverlappingTemplate

2	0	3	0	1	2	1	1	1	0	0.437274	11/11	NonOverlappingTemplate
1	0	1	1	1	0	2	1	1	3	0.637119	11/11	NonOverlappingTemplate
1	3	1	1	0	1	2	2	0	0	0.437274	10/11	NonOverlappingTemplate
1	0	1	1	0	2	2	2	1	1	0.834308	10/11	NonOverlappingTemplate
1	1	1	1	2	3	1	0	0	1	0.637119	11/11	NonOverlappingTemplate
2	0	2	0	1	2	1	1	1	1	0.834308	11/11	NonOverlappingTemplate
1	0	0	2	1	2	0	0	0	5	0.006196	11/11	NonOverlappingTemplate
0	0	2	0	4	1	0	3	0	1	0.025193	11/11	NonOverlappingTemplate
1	1	1	0	1	2	2	0	2	1	0.834308	11/11	NonOverlappingTemplate
1	2	0	0	1	0	2	4	1	0	0.090936	11/11	NonOverlappingTemplate
2	0	0	2	1	2	2	2	0	0	0.437274	11/11	NonOverlappingTemplate
1	0	1	0	1	2	0	2	2	2	0.637119	11/11	NonOverlappingTemplate
3	0	0	4	1	1	1	0	0	1	0.048716	10/11	NonOverlappingTemplate
2	0	1	1	2	0	2	2	1	0	0.637119	11/11	NonOverlappingTemplate
0	1	0	3	0	1	0	1	2	3	0.162606	11/11	NonOverlappingTemplate
1	1	0	0	2	1	2	1	1	2	0.834308	11/11	NonOverlappingTemplate
2	1	1	1	1	0	1	2	0	2	0.834308	10/11	NonOverlappingTemplate
1	2	0	0	2	1	0	1	3	1	0.437274	11/11	NonOverlappingTemplate
1	0	2	0	0	2	2	1	2	1	0.637119	11/11	NonOverlappingTemplate
1	0	0	2	1	1	1	1	2	2	0.834308	11/11	NonOverlappingTemplate
0	1	1	3	0	2	1	1	0	2	0.437274	11/11	NonOverlappingTemplate
2	0	2	2	1	1	2	0	0	1	0.637119	9/11	NonOverlappingTemplate
1	1	0	1	2	2	1	0	1	2	0.834308	11/11	NonOverlappingTemplate
2	3	1	2	1	0	0	0	1	1	0.437274	10/11	NonOverlappingTemplate
0	0	2	0	0	1	2	2	2	2	0.437274	11/11	NonOverlappingTemplate
1	3	1	0	1	2	1	1	1	0	0.637119	10/11	NonOverlappingTemplate
0	2	0	2	2	1	2	0	0	2	0.437274	11/11	NonOverlappingTemplate
1	0	1	1	0	1	0	3	3	1	0.275709	11/11	NonOverlappingTemplate
2	0	1	1	0	2	2	1	0	2	0.637119	11/11	NonOverlappingTemplate
2	1	0	0	1	3	4	0	0	0	0.025193	11/11	NonOverlappingTemplate
1	1	2	1	3	2	0	1	0	0	0.437274	11/11	NonOverlappingTemplate
1	0	1	1	2	1	0	1	1	3	0.637119	11/11	NonOverlappingTemplate
2	1	1	0	2	1	1	1	1	1	0.964295	11/11	NonOverlappingTemplate
1	1	0	3	0	1	1	1	0	3	0.275709	10/11	NonOverlappingTemplate
2	1	0	1	1	0	1	2	2	1	0.834308	11/11	NonOverlappingTemplate
0	2	0	2	2	4	0	0	0	1	0.048716	11/11	NonOverlappingTemplate
0	3	2	0	1	0	2	2	1	0	0.275709	11/11	NonOverlappingTemplate
1	2	1	1	0	1	3	0	0	2	0.437274	11/11	NonOverlappingTemplate
1	0	0	1	0	1	3	3	2	0	0.162606	11/11	NonOverlappingTemplate
0	0	1	3	2	1	2	0	2	0	0.275709	11/11	NonOverlappingTemplate
0	0	1	2	0	0	2	1	2	3	0.275709	11/11	NonOverlappingTemplate
2	1	2	2	0	0	2	0	0	2	0.437274	11/11	NonOverlappingTemplate

1	0	1	0	1	2	1	1	2	2	0.834308	10/11	NonOverlappingTemplate
1	0	2	1	2	3	1	1	0	0	0.437274	11/11	NonOverlappingTemplate
3	0	1	3	0	1	1	0	0	2	0.162606	11/11	NonOverlappingTemplate
2	1	2	1	0	1	0	1	1	2	0.834308	9/11	NonOverlappingTemplate
2	0	0	3	3	0	0	2	1	0	0.090936	11/11	NonOverlappingTemplate
3	3	0	0	1	1	1	0	0	2	0.162606	10/11	NonOverlappingTemplate
1	1	3	2	0	1	0	0	2	1	0.437274	11/11	NonOverlappingTemplate
0	1	1	0	2	3	2	0	1	1	0.437274	11/11	NonOverlappingTemplate
1	0	0	2	4	0	2	0	1	1	0.090936	11/11	NonOverlappingTemplate
0	2	0	3	0	1	3	0	1	1	0.162606	11/11	NonOverlappingTemplate
0	0	1	3	2	2	0	0	2	1	0.275709	11/11	NonOverlappingTemplate
1	1	0	0	1	1	3	0	2	2	0.437274	11/11	NonOverlappingTemplate
1	0	0	4	1	1	1	1	0	2	0.162606	11/11	NonOverlappingTemplate
1	1	0	0	2	3	1	1	0	2	0.437274	11/11	NonOverlappingTemplate
0	3	0	2	1	0	2	0	2	1	0.275709	11/11	NonOverlappingTemplate
2	1	0	1	1	2	0	2	1	1	0.834308	11/11	NonOverlappingTemplate
2	0	1	1	2	1	1	1	2	0	0.834308	11/11	NonOverlappingTemplate
1	0	1	2	0	1	2	0	1	3	0.437274	10/11	NonOverlappingTemplate
1	2	2	0	0	2	1	0	1	2	0.637119	10/11	NonOverlappingTemplate
0	2	1	0	2	0	1	1	2	2	0.637119	11/11	NonOverlappingTemplate
0	2	1	0	1	3	3	0	1	0	0.162606	11/11	NonOverlappingTemplate
1	0	1	0	0	0	1	3	3	2	0.162606	11/11	NonOverlappingTemplate
2	0	1	2	0	1	2	0	0	3	0.275709	11/11	NonOverlappingTemplate
3	1	2	0	2	1	1	0	0	1	0.437274	11/11	NonOverlappingTemplate
0	1	0	0	0	1	0	4	1	4	0.006196	11/11	NonOverlappingTemplate
1	2	0	0	0	0	3	2	1	2	0.275709	11/11	NonOverlappingTemplate
3	3	1	0	0	0	2	2	0	0	0.090936	11/11	NonOverlappingTemplate
2	2	3	0	2	0	0	0	0	2	0.162606	11/11	NonOverlappingTemplate
1	0	1	1	0	1	1	0	3	3	0.275709	11/11	NonOverlappingTemplate
0	1	1	2	2	0	0	2	2	1	0.637119	11/11	NonOverlappingTemplate
1	1	2	1	0	2	1	1	2	0	0.834308	10/11	NonOverlappingTemplate
2	2	0	3	0	0	2	1	1	0	0.275709	10/11	NonOverlappingTemplate
0	1	3	2	0	2	0	1	2	0	0.275709	11/11	NonOverlappingTemplate
1	0	0	1	1	1	4	1	1	1	0.275709	11/11	NonOverlappingTemplate
0	0	1	1	1	3	2	0	1	2	0.437274	11/11	NonOverlappingTemplate
1	0	1	0	2	2	3	1	0	1	0.437274	11/11	NonOverlappingTemplate
3	1	1	0	2	1	0	2	0	1	0.437274	10/11	NonOverlappingTemplate
1	0	0	0	3	3	2	1	0	1	0.162606	11/11	NonOverlappingTemplate
1	1	0	0	1	2	1	2	1	2	0.834308	11/11	NonOverlappingTemplate
1	0	0	1	1	0	1	2	2	3	0.437274	11/11	NonOverlappingTemplate
0	0	3	0	2	0	0	1	2	3	0.090936	11/11	NonOverlappingTemplate

0	1	1	1	0	1	0	3	1	3	0.275709		11/11		NonOverlappingTemplate
1	2	0	0	3	0	0	2	1	2	0.275709		11/11		NonOverlappingTemplate
1	1	0	1	2	0	1	3	1	1	0.637119		11/11		NonOverlappingTemplate
1	1	0	3	1	1	1	0	1	2	0.637119		11/11		NonOverlappingTemplate
1	1	0	1	2	1	1	1	1	2	0.964295		10/11		NonOverlappingTemplate
4	1	0	0	1	0	2	2	0	1	0.090936		10/11		NonOverlappingTemplate
1	2	1	0	1	2	1	1	1	1	0.964295		11/11		NonOverlappingTemplate
0	1	0	1	2	0	0	3	1	3	0.162606		11/11		NonOverlappingTemplate
3	2	0	0	0	1	2	1	1	1	0.437274		11/11		NonOverlappingTemplate
0	1	1	1	2	0	1	1	1	3	0.637119		11/11		NonOverlappingTemplate
1	2	2	1	2	1	1	1	0	0	0.834308		11/11		NonOverlappingTemplate
0	2	2	1	1	1	1	1	1	1	0.964295		11/11		NonOverlappingTemplate
1	0	3	1	1	1	1	0	1	2	0.637119		11/11		NonOverlappingTemplate
0	0	0	0	2	3	1	2	1	2	0.275709		11/11		NonOverlappingTemplate
0	1	2	0	1	1	1	3	1	1	0.637119		11/11		NonOverlappingTemplate
1	0	1	2	2	1	1	1	0	2	0.834308		11/11		NonOverlappingTemplate
1	1	0	5	1	2	0	0	1	0	0.012650		11/11		NonOverlappingTemplate
1	1	0	1	2	3	1	1	0	1	0.637119		11/11		NonOverlappingTemplate
2	0	4	1	1	1	1	0	1	0	0.162606		10/11		NonOverlappingTemplate
1	3	0	1	0	3	0	2	0	1	0.162606		11/11		NonOverlappingTemplate
2	1	1	2	0	0	2	1	1	1	0.834308		11/11		NonOverlappingTemplate
2	0	0	2	0	1	1	1	1	3	0.437274		10/11		NonOverlappingTemplate
1	0	0	0	2	2	1	2	3	0	0.275709		11/11		NonOverlappingTemplate
3	0	0	3	1	2	1	1	0	0	0.162606		10/11		NonOverlappingTemplate
1	0	1	1	1	3	1	1	0	2	0.637119		11/11		NonOverlappingTemplate
1	0	3	0	1	1	0	2	2	1	0.437274		11/11		NonOverlappingTemplate
0	0	0	3	1	1	2	0	3	1	0.162606		11/11		NonOverlappingTemplate
2	1	0	0	2	0	0	2	0	4	0.048716		11/11		NonOverlappingTemplate
1	0	1	2	0	1	2	1	2	1	0.834308		11/11		NonOverlappingTemplate
0	1	0	1	2	0	1	4	0	2	0.090936		11/11		NonOverlappingTemplate
0	2	1	0	1	1	1	2	0	3	0.437274		11/11		NonOverlappingTemplate
1	0	2	1	0	1	1	1	4	0	0.162606		11/11		NonOverlappingTemplate
1	1	2	0	1	2	0	2	0	2	0.637119		11/11		NonOverlappingTemplate
1	0	0	2	4	0	0	2	1	1	0.090936		11/11		NonOverlappingTemplate
1	2	1	1	0	1	1	2	1	1	0.964295		11/11		NonOverlappingTemplate
2	2	2	0	3	0	0	1	1	0	0.275709		11/11		NonOverlappingTemplate
1	0	1	1	1	0	2	2	3	0	0.437274		10/11		NonOverlappingTemplate
2	0	0	3	3	0	0	2	1	0	0.090936		11/11		NonOverlappingTemplate
0	1	2	1	0	2	1	1	3	0	0.437274		11/11		OverlappingTemplate
11	0	0	0	0	0	0	0	0	0	0.000000	*	0/11	*	Universal
9	1	1	0	0	0	0	0	0	0	0.000000	*	8/11	*	ApproximateEntropy

0	0	0	0	0	0	0	0	0	0	----	------	RandomExcursions
0	0	0	0	0	0	0	0	0	0	----	------	RandomExcursions
0	0	0	0	0	0	0	0	0	0	----	------	RandomExcursions
0	0	0	0	0	0	0	0	0	0	----	------	RandomExcursions
0	0	0	0	0	0	0	0	0	0	----	------	RandomExcursions
0	0	0	0	0	0	0	0	0	0	----	------	RandomExcursions
0	0	0	0	0	0	0	0	0	0	----	------	RandomExcursions
0	0	0	0	0	0	0	0	0	0	----	------	RandomExcursions
0	0	0	0	0	0	0	0	0	0	----	------	RandomExcursionsVariant
0	0	0	0	0	0	0	0	0	0	----	------	RandomExcursionsVariant
0	0	0	0	0	0	0	0	0	0	----	------	RandomExcursionsVariant
0	0	0	0	0	0	0	0	0	0	----	------	RandomExcursionsVariant
0	0	0	0	0	0	0	0	0	0	----	------	RandomExcursionsVariant
0	0	0	0	0	0	0	0	0	0	----	------	RandomExcursionsVariant
0	0	0	0	0	0	0	0	0	0	----	------	RandomExcursionsVariant
0	0	0	0	0	0	0	0	0	0	----	------	RandomExcursionsVariant
0	0	0	0	0	0	0	0	0	0	----	------	RandomExcursionsVariant
0	0	0	0	0	0	0	0	0	0	----	------	RandomExcursionsVariant
0	0	0	0	0	0	0	0	0	0	----	------	RandomExcursionsVariant
0	0	0	0	0	0	0	0	0	0	----	------	RandomExcursionsVariant
0	0	0	0	0	0	0	0	0	0	----	------	RandomExcursionsVariant
0	0	0	0	0	0	0	0	0	0	----	------	RandomExcursionsVariant
0	0	0	0	0	0	0	0	0	0	----	------	RandomExcursionsVariant
0	0	0	0	0	0	0	0	0	0	----	------	RandomExcursionsVariant
0	0	0	0	0	0	0	0	0	0	----	------	RandomExcursionsVariant
0	0	0	0	0	0	0	0	0	0	----	------	RandomExcursionsVariant
3	0	0	0	2	2	1	1	1	1	0.437274	9/11	Serial
3	0	1	0	1	1	2	1	0	2	0.437274	9/11	Serial
1	0	2	1	2	0	2	2	1	0	0.637119	10/11	LinearComplexity

- -
The minimum pass rate for each statistical test with the exception of the random excursion (variant) test is approximately = 9 for a sample size = 11 binary sequences.

The minimum pass rate for the random excursion (variant) test is undefined.

For further guidelines construct a probability table using the MAPLE program provided in the addendum section of the documentation.
- -

Supplementary Discussions

The discussion of Fig. 3 and simulation method

The abrupt breaking of the CF is mainly attributed to the Joule heating effect. When the Joule heating is strong enough, the CF will be broken in to multiple segments. Therefore, the effective length of the broken CF is calculated as the part of the CF which is connected to one boundary of SnO_x layer. Meanwhile, within the relaxation time of Joule heating, the relatively high temperature will accelerate the movement of oxygen ions and this will make the CF grow from the effective broken length.

After two sets at LRS, "$V-V_1$" will approximately be equal to "V_2" and less than V/2 because the SnSe layer will occupy some voltage potential. The Joule heating generated along the CF will be the major reason of the reset behavior in metal-oxide RRAM[2]. The strength of Joule heating has a positive correlation with the product of current and voltage across the RRAM. When both CFs are formed across the two SnO_x layers, a high current will traverse both RRAMs. As the voltage increases, Joule heating effect is high enough to break the CF in the upper layer. This causes the voltage in the upper layer to increase and the voltage in the lower layer to decrease suddenly. Current will consequently decrease and Joule heating will weaken. Due to the relatively high electric field existing in the upper layer, the filament will grow towards the top electrode. When the voltage and total current become high, a competition will occur between the CF breakage caused by Joule heating and the CF forming induced by electric field. Initially, the Joule heating dominates over the effect caused by the electric field and causes the filament to be broken again before the

filament can form. Then the electric field makes the CF grow again. As the voltage and current are continuously increased, the CF begins to grow near the top; however, the Joule heating is so strong that both filaments break. If we continue to increase the voltage, the Joule heating will be more significant and strongly dominate over the effect of the electric field. As a result, the upper filament will break twice and it's very hard to make the device set again after increasing voltage to a higher level.

The driving force for CF in equation (14b) is Joule heating. The detail of Joule heating effect is discussed above. The temperature contains an exponential term, which indicates that high temperature leads to high speed of CF forming and rupture. But we also have to consider the free parameters -- when the Joule heating is strong enough, for unipolar RRAM, the sign of *s* will be reversed and become the negative value, which drives the RRAM from low resistive state to high resistive state.

The equations used in the simulation are mainly composed of two parts: (1) Current calculation with a fixed CF at certain time point, and (2) the CF length determined by electrical field and Joule heating varying with time. In the current calculation part (1), as the metal oxide (SnO_x) is used as a resistive switching layer, the CF made of vacancies and electron tunneling model inside the oxide layer is used to analyze its dynamic behavior. According to Ref. 3 and Ref. 4, Equation 9(a) and 9(b) are used to calculate the tunneling current in the gap between the top electrode and top part of CF in the insulation layer. As the CF length (l) is a function of time (t), we can expand the function $l(t+\Delta t)$ by Taylor formula at the point $t=t$, which is:

$$l(t+\Delta t) = l(t) + l'(t)\Delta t + \frac{l''(t)}{2!}(\Delta t)^2 + \frac{l'''(t)}{3!}(\Delta t)^3 + o((\Delta t)^4) \qquad (1)$$

During the simulation, Δt is very small and the order of Δt higher than one can be neglected, therefore $l(t+\Delta t)$ can be written as equation (13). As to the speed of filament growth ($\frac{dl}{dt}$), it is mainly determined by the "drifting" speed of leading ions, which is further related to the time taken for the ions to hop over an energy barrier. The mathematic model is shown as Equation 14a[5]. However, if h-l is very small, the filament speed calculation will cause non-physical results and overestimate the growth speed. Meanwhile, other processes such as metal atom oxidization rate will dominate it when the gap is tiny enough. Therefore the growth speed of CF can be rewritten as Equation 14b[4].

Calculate the theoretical probabilities and validation of the Markov chain

Equation (1) is the definition of the Markov chain, which means that the probability of the current state is only dependent on the last state and is irrelevant to the other past states. In our device, there are three stages and nine stage-transfer conditions(i->i, i->ii, i->iii, ii->i, ii->ii, ii->iii, iii->i, iii->ii, iii->iii). After careful analysis of the data, we find that all nine stage-transfer probabilities will be convergent to nine steady values as the cycles increase (Figure 4d). This means that all transfer probabilities between the current stage and the last stage is constant and not variable with other past stages. Based on Simpson's law of large numbers[6], for any given positive real number ε, which can be chosen as close to zero as possible, the sequence of state variable satisfies the following equation:

$$\lim_{n\to\infty} P\left(\left|\frac{1}{n}\sum_{i=1}^{n} a_i - \mu\right| < \varepsilon\right) = 1 \qquad (2)$$

Where $P(.)$ is the probability and μ is the expectation. From the law, we can see that if the experimental testing number (n) is large enough, the expected probability is equal to the convergence value we have obtained. In our device, $\hat{p}_{ji}^{m}$ can be written as

$$\hat{p}_{ji}^{m} = \frac{\sum_{k=1}^{m} I(i->j\,|\,i_0, j_0)}{\sum_{k=1}^{m} I(i\,|\,i_0)} = \frac{\sum_{k=1}^{m} I(i->j\,|\,i_0, j_0)}{n} = \frac{\sum_{i=1}^{m} a_i}{n} \qquad (3)$$

And $\mu = p_{ji}\times 1 + (1-p_{ji})\times 0 = p_{ji}$, Therefore $p_{ji} = \lim_{m\to\infty} \hat{p}_{ji}^{m}$. As the $\lim_{m\to\infty} \hat{p}_{ji}^{m}$ exists and is constant for all transfer stages, this chain is a Markov chain and $\hat{p}_{ji}^{m}$ can be regarded as the theoretical transfer probability from stage i to j if the m is large enough. In order to eliminate the random fluctuation of $\hat{p}_{ji}^{m}$, we averaged the last 30 $\hat{p}_{ji}^{m}$ to use as p_{ji} and mismatch error is calculated between the $\hat{p}_{ji}^{m}$ and p_{ji}. As to the error rate of the fixed-probability random number, due to state transition matrix can be obtained via above calculation of p_{ji}. The theoretical probabilities of three random numbers can be acquired via equation (7) in the manuscript. These values are calculated according to the mathematical property of the Markov chain but not extracted from the experimental data directly.

From Figure 4d, we can see that the percentage of all states transfer is convergent to a constant value, which is equal to theoretical value of percentage according to Simpson's law of large numbers discussed above. This constant value can be extracted from the Figure 4d and the percentage of each state transfer (C_{ji}) can be written as

table 2.

In Markov chain, the sum of transfer probability from certain state to all other state (including itself) is definitely equal to 1, which means:

$$\sum_{j} p_{ji} = 1 \qquad (4)$$

For C_{ji}, it's equivalent to the possibility which the current stage is j and last stage is i, which means that $C_{ji} = P(X_{n+1} = j, X_n = i)$. Meanwhile, p_{ji} can be written as $P(X_{n+1} = j \mid X_n = i)$. Now taking the Bayes formula, which can be written as,

$$P(X_{n+1} = j, X_n = i) = P(X_{n+1} = j \mid X_n = i)P(X_n = i) = P(X_{n+1} = j \mid X_n = i)\sum_{k} P(X_n = i \mid X_{n-1} = k)$$

and simplifying it, the p_{ji} is equal to $\frac{C_{ji}}{\sum_{k} C_{jk}}$, which is the element of transition probability matrix A (A_{ji}). Based on the analysis above, the transition probability matrix A is equal to the following matrix.

$$A = \begin{bmatrix} 0.433 & 0.250 & 0.317 \\ 0.243 & 0.390 & 0.367 \\ 0.268 & 0.446 & 0.286 \end{bmatrix} \qquad (5)$$

The theoretical random number probability is obtained via transition probability matrix and the Markov Chain unique property (Equation (7)) (see Supplementary Figure 15).

It is noticed that the transition probability matrix is estimated by the state-transition data (such as i→ii,ii→ii, iii→ii) where the sequence order of states matters. However, when figuring out the probability frequency of single states, only the total number of the various states is considered (and not the order). Therefore the data used for estimating the theoretical value and keeping statistics of a certain state probability

frequency is different, and cannot simply be regarded as data self-comparison. On the other hand, the low error rate can show that our device has good Markov chain properties. This is further supported by the fact that the experimental results have a good consistency with the probability estimation (as calculated using the unique mathematical characteristics of the Markov chain).

NIST test

We have tried to verify the data for the NIST test. As the NIST test requires a large amount of data, therefore, the data has been generated from ~2.2×10^5 cycles of hardware operation using the sawtooth wave test on the SnSe RRAM. What's more, as the NIST test measures the randomness of '0'/'1' sequence under the condition of 50% generated probability, testing results of the proposed device which contains three states cannot directly be used in the NIST test. Therefore, we assign the state III to '0' (state I) or '1'(state II) according to the following rules: if the frequency number of '0' or '1' in the sequence before state iii is less than 50%, the states iii is assigned to it. This operation can make sure the final probability of both '0' and '1' is equal to 50%. Prior to the NIST test, for the true random number, post-processing is required to eliminate the physical effects which causes deviation and correlation in generated sequences[6]. For our post-processing, the resilient function is used to deal with raw data. After post-processing, 112941 '0'/'1' random numbers was obtained. The bit-stream length is set to 10,000 and group number is 11 on NIST configuration. The results are shown in Supplementary Table 3.

From the NIST result, we can see that apart from the ‘Universal’ and ‘ApproximateEntroy’ items, all other testable items have passed the NIST test. Moreover, for the ‘ApproximateEntroy’ item, it has nearly passed (9/11 is the minimum pass rate, whereas our random number is 8/11). The final NIST report reveals that the random numbers generated by our devices have overall good randomness and the potential to be applied in industry in the future. For the ‘Universal’ item which did not pass, it is because this test requires a longer sequence of bits (the number of minimum bits is 387840).

Supplementary References

1 Yao, J. *et al.* Highly transparent nonvolatile resistive memory devices from silicon oxide and graphene. *Nat. Commun.* **3**, 1101 (2012).

2 Wong, H. S. P. *et al.* Metal-Oxide RRAM. *Proc. IEEE* **100**, 1951-1970 (2012).

3 Stratton, R. Volt-Current Characteristics for Tunneling Through Insulating Films. *J. Phys. Chem. Solids* **23**, 1177-1190 (1962).

4 Sheridan, P. *et al.* Device and SPICE modeling of RRAM devices. *Nanoscale* **3**, 3833-3840 (2011).

5 Strukov, D. B. & Williams, R. S. Exponential ionic drift: fast switching and low volatility of thin-film memristors. *Appl. Phys. A: Mater. Sci. Process.* **94**, 515-519 (2009).

6 Golic, J. D. New methods for digital generation and postprocessing of random data. *IEEE Trans. Comput.* **55**, 1217-1229 (2006).
